# An interpretable XAI deep EEG model for schizophrenia diagnosis using feature selection and attention mechanisms

**DOI:** 10.3389/fonc.2025.1630291

**Published:** 2025-07-22

**Authors:** Ahmad Almadhor, Stephen Ojo, Thomas I. Nathaniel, Shtwai Alsubai, Abdullah Alharthi, Abdullah Al Hejaili, Gabriel Avelino Sampedro

**Affiliations:** ^1^ Department of Computer Engineering and Networks, College of Computer and Information Sciences, Jouf University, Sakaka, Saudi Arabia; ^2^ Department of Electrical and Computer Engineering, College of Engineering, Anderson University, Anderson, SC, United States; ^3^ School of Medicine Greenville, University of South Carolina, Greenville, SC, United States; ^4^ College of Computer Engineering and Sciences, Prince Sattam bin Abdulaziz University, AlKharj, Saudi Arabia; ^5^ Department of Electrical Engineering, King Khalid University, Abha, Saudi Arabia; ^6^ Computer Science Department, Faculty of Computers & Information Technology, University of Tabuk, Tabuk, Saudi Arabia; ^7^ School of Management and Information Technology, De La Salle-College of Saint Benilde, Manila, Philippines

**Keywords:** schizophrenia, electroencephalography (EEG), SHAP, LIME, feature selection, SMOTE

## Abstract

**Introduction:**

Schizophrenia is a severe psychological disorder that significantly impacts an individual’s life and is characterized by abnormalities in perception, behavior, and cognition. Conventional Schizophrenia diagnosis techniques are time- consuming and prone to error. The study proposes a novel automated technique for diagnosing Schizophrenia based on electroencephalogram (EEG) sensor data, aiming to enhance interpretability and prediction performance.

**Methods:**

This research utilizes Deep Learning (DL) models, including the Deep Neural Network (DNN), Bi-Directional Long Short-Term Memory-Gated Recurrent Unit (BiLSTM- GRU), and BiLSTM with Attention, for the detection of Schizophrenia based on EEG data. During preprocessing, SMOTE is applied to address the class imbalance. Important EEG characteristics that influence model decisions are highlighted by the interpretable BiLSTM-Attention model using attention weights in conjunction with SHAP and LIME explainability tools. In addition to fine-tuning input dimensionality, F-test feature selection increases learning efficiency.

**Results:**

Through the integration of feature importance analysis and conventional performance measures, this study presents valuable insights into the discriminative neurophysiological patterns associated with Schizophrenia, advancing both diagnostic and neuroscientific expertise. The experiment’s findings show that the BiLSTM with attention mechanism model provides and accuracy of 0.68%.

**Discussion:**

The results show that the recommended approach is useful for Schizophrenia diagnosis.

## 1 Introduction

Schizophrenia is a severe brain disorder that significantly impacts a person’s memory, cognition, comprehension, behavior, and communication (1). This chronic mental disease impacts an individual’s entire quality of life by having a substantial impact on lifestyle, relationships, and profession (2). Disturbingly, 20-40% of people with Schizophrenia have attempted suicide at least once, and a significant portion of them have difficulty functioning in professional settings (3). The World Health Organization (WHO) estimates that 20 million people worldwide have this mental illness (4). Furthermore, the WHO has also underlined that Schizophrenia is curable, emphasizing the need for an accurate and timely diagnosis for patients’ recovery and overall health.

The subjective assessment of behavioral symptoms by clinicians, such as continuous functional deterioration, delusions, hallucinations, and disordered speech or thought patterns, is currently a significant component of the diagnosis of Schizophrenia. Standardized criteria, such as those listed in the DSM-5, are typically used to guide these assessments; however, ultimately, the clinician’s opinion and experience are what matter most. Due to this, the diagnostic process is often susceptible to bias, inconsistency, and misdiagnosis, particularly in atypical or early-stage situations. The absence of a globally recognized, objective clinical diagnosis for Schizophrenia continues to be a significant obstacle to prompt and precise diagnosis. To address these limitations, biological data-based techniques that are repeatable, automated, and dependable are needed. One advantage of such a system is that it can be applied in a wide range of situations without requiring experts with extensive experience. Electroencephalograms (EEGs) are a potent tool in the diagnosis of mental disorders because they can accurately assess the state of the brain (5, 6). Clinical applications have made extensive use of it (7). Moreover, EEG is commonly used due to its portability, non-invasiveness, ease of setup, and high temporal resolution (8). EEGs in source localizations have been used to successfully diagnose several brain disorders, including epilepsy, Schizophrenia, and Parkinson’s disease (9, 10). For instance, identifying active areas linked to spikes is a crucial difficulty in epilepsy research. However, accurate source localizations are essential for TMS treatment techniques for mental diseases like schizophrenia (11). According to recent research, human-computer interface technologies have been greatly improved by the combination of AI and EEG. AI-based EEG systems prioritize robustness and interpretability over traditional EEG systems. These studies provide the first thorough survey, classifying robustness against noise and artifacts, human variety, inconsistent data collection, adversarial threats, and interpretability into backpropagation, perturbation, and intrinsically interpretable techniques. It discusses possible potential paths and brings attention to unresolved problems (12, 13).

Motivation: Numerous machine learning (ML) approaches based on electroencephalogram (EEG) data have been investigated for feature extraction, relevant feature selection, and classification to automate the recognition of schizophrenia (14). However, the advent of deep learning (DL) models has raised much attention lately since they offer a more potent and automated way to discover discriminative patterns directly from unprocessed or limited processed data (15). Convolutional neural networks (CNNs), in particular, which are extremely significant for classification problems, including in clinical neuroscience, can automatically extract hierarchical features, in contrast to traditional attribute-based approaches. According to Afzali et al. (16), these methods replicate how the human brain processes information and generates decision-making patterns. Developments in Neural Network (NN) architecture design and training have transformed deep learning (DL), allowing researchers to focus on challenges in learning that were previously difficult. In the context of diagnosing Schizophrenia using EEG, CNNs’ capacity to capture temporal and spatial correlations in brain signal patterns has demonstrated significant promise (17). Furthermore, deep representations for classifying Schizophrenia in fMRI data have been captured using advanced network topologies, such as 3D CNNs in combination with autoencoders (18). There are still major challenges to be solved, however. Current DL and ML models typically have low generalizability due to the use of small and unbalanced datasets, which is problematic because clinical trust and acceptance rely on clear decision-making procedures. In this study, we present an interpretable deep-learning pipeline for utilizing EEG data in the detection of Schizophrenia. Our method combines several elements: a strong preprocessing pipeline that prepares the raw EEG signals by segmenting, normalizing, and removing artifacts; carefully chosen DL architectures, such as a baseline DNN, a hybrid BiLSTM-GRU model to capture temporal dependencies, and a BiLSTM-Attention model that improves feature relevance through attention mechanisms; synthetic minority over-sampling technique (SMOTE) to address class imbalance; and explainable AI (XAI) techniques SHAP, LIME, and Integrated Gradients to visualize and interpret the model’s predictions. In addition to improving classification accuracy, the result provides the degree of interpretability required for practical healthcare implementation.

This research enables a more accurate and efficient diagnosis of Schizophrenia. The following lists the main findings and contributions of the research.

- The study develops and evaluates three deep learning models: DNN, BiLSTM-GRU, and BiLSTM, with attention to the objective of detecting Schizophrenia using EEG. Each architecture captures distinct spatial and temporal patterns in the data. The BiLSTM-Attention model outperforms the other models by effectively focusing on the most informative EEG segments, emphasizing the significance of attention processes in clinical applications.
- The implementation of an interpretable BiLSTM-Attention model is improved with SHAP and LIME tools, which provide concise rationales for the model’s conclusions. Consequently, key EEG features that are most discriminative in identifying Schizophrenia can be discovered, providing clinically significant insights.
- This study uses SMOTE to handle class imbalance and F-test feature selection to improve model efficiency. By integrating feature importance analysis with conventional performance measurements, the evaluation methodology provides a thorough and accessible assessment of model performance, along with the underlying neurophysiological processes that contribute to Schizophrenia.

The notions used throughout the paper are shown in **Table 1**. The following section provides further structure for the paper. Section 2 details the background data on schizophrenia diagnosis. Section 3 describes the suggested approach. In Section 4, the effectiveness of the suggested approach is evaluated and compared to the baseline methods. Section 5 concludes the entire paper and provides recommendations for further research.

**Table 1:** Abbreviations.

Abbreviation	Description
AI	Artificial Intelligence
ANN	Artificial Neural Network
NB	Naive Bayes
CNN	Convolutional Neural Network
DL	Deep Learning
DNN	Deep Neural Network
DT	Decision Tree
DWT	Discrete Wavelet Transform
EEG	Electroencephalogram
GRU	Gated Recurrent Unit
KNN	K-Nearest Neighbor
LSTM	Long Short Term Memory
LIME	Local Interpretable Model-Agnostic Explanations
ML	Machine Learning
MAO	Mutation-boosted Archimedes Optimization
NB	Naive Bayes
RF	Random Forest
RNN	Recurrent Neural Network
RT	Random Tree
SZ	Schizophrenia
SHAP	Shapley Additive Explanations
SMOTE	Synthetic Minority Oversampling Technique
STRGCN	Spatial-Temporal Residual Graph Neural Convolutional Network
SVM	Support Vector Machine
WHO	World Health Organization
XAI	Explainable AI

## 2 Related work

This section details the background of Schizophrenia Diagnosis using different ML and DL techniques. In research (19), the author investigates ML approaches to categorize individuals with schizophrenia proneness levels according to demographic and behavioral features such as age, movement, pain, slowness, exhaustion, and cleanliness. The dataset comprises 1,000 samples, divided into five categories of proneness. Logistic Regression, Support Vector Machine (SVM), Gradient Boosting, and Decision Tree classifiers were all assessed in the study. Logistic Regression yielded the highest accuracy, 94.2%. In (20), the author developed a diagnostic tool using ML software to extract known dysregulated miRNAs from the List literature that have been demonstrated to have potential as biomarkers. To distinguish SCZ-associated miRNA biomarkers from those chosen at random, a method was developed. After validation, the model’s accuracy on an independent test set was 88.88%, having achieved a peak of 94.32% with the sequential classifier. In the study (21), the authors employed nine global centers to train and test the generalizability of the 3D ResNet model. Using a leave-one-center-out validation technique, the model achieved an 82% classification performance on all datasets originating from various countries. The study identified significant abnormalities between healthy controls and those with Schizophrenia in the thalamus, pallidum, and inferior frontal gyrus. An anatomical atlas was utilized to enhance the SHAP permutation explainer’s therapeutic applicability by providing accurate neuroanatomical and functional interpretations.

In research (22), the author presents a novel approach for optimizing preprocessing stages for better EEG data quality by using the Mutation-boosted Archimedes Optimization (MAO) algorithm for schizophrenia Detection. Using a Long Short-Term Memory (LSTM) network based on Convolutional Neural Networks (CNNs), the spatial and temporal patterns are retrieved from multichannel EEG data. Experimental testing on various datasets confirms the efficacy of the suggested method. The method outperforms current methods with 98.2% accuracy. In the study (23), the authors present a spatial-temporal residual graph convolutional neural network (STRGCN)- based classification method for SZ patients. The model primarily utilizes single-channel temporal convolution and spatial graph convolution to collect temporal and spatial frequency data, respectively and then combines these two for classification learning. Extensive experiments were conducted on the author’s dataset and the publicly accessible Zenodo dataset. The classification accuracy of the two datasets using the recommended method was 85.44% and 96.32%, respectively. In the study (24), the author presents a novel approach to SZ diagnosis that combines adaptive statistical parametric mapping, level analysis, and seed-based voxel activation. The study incorporates pre-trained deep learning models (DLMs) from the ImageNet dataset, including VGG-16, ResNet50, MobileNet, and a recently created simplified DLM called SZ-Net. The results demonstrate that SZ-Net is capable of correctly classifying fMRI scans, with an excellent 10-fold validation classification accuracy of 99.24%.

In research (25), the author presents a novel method for automatically identifying Schizophrenia by fusing fuzzy logic with deep convolution models. CNN uses type-2 fuzzy notions for its activation functions to handle uncertainty. Generative adversarial networks are utilized to process data, thereby reducing overfitting and enhancing diagnostic accuracy. This technique is evaluated using electroencephalogram signals from both healthy controls and patients with Schizophrenia, showing a remarkable 99.05% accuracy rate in differentiating between the two groups. Additionally, the suggested method is externally validated using a database of unseen electroencephalograms, yielding a 96.42% diagnosis accuracy, 100% sensitivity, and 92.85% specificity. In research (26), the authors present the usage of deep learning and EEG connection measurements, which indicates potential in accurately identifying brain disorders, including Schizophrenia and Alzheimer’s. Using coherence and PLV analysis, the system detected and quantified disease-specific changes in brain patterns with 94% accuracy for AD and 91% accuracy for Schizophrenia. While maintaining low diagnostic costs and employing non-invasive techniques, the combination of deep learning and EEG technology demonstrates improved accuracy and a more straightforward implementation in clinics.

In research (27), the author uses short-duration electrocardiogram (ECG) signals captured using a lowcost wearable device to develop and evaluate an automated classification technique for bipolar illness and Schizophrenia. The study analyzed R-R interval windows extracted from brief ECG recordings and performed classification tests using machine learning (ML) techniques. There were 60 participants in the study: 30 people with bipolar illness or Schizophrenia and 30 control subjects. The study evaluated numerous ML models, achieving classification accuracy of 83% for the 5-fold cross-validation and 80% for the leave-one-out scenario. In research (28), the authors present five new frameworks based on machine learning (ML) and deep learning (DL) for detecting Schizophrenia from EEG signals. The present study is among the first to diagnose Schizophrenia using a 2D representation of entropy features taken from DWT and MEMD-processed signals. In the proposed DWT-based framework, the classification performance of different CNN models ranges from 57.14% to 86.59%. Likewise, CNN’s performance for the MEMD-based framework ranges from 54.19 to 85.19%. The maximum accuracy attained by the basic CNN for DWT and MEMD-based complexity features was 86.59% and 85.19%, respectively. The FFNN model produces a remarkable 88.18% classification accuracy. Lastly, the fifth framework further improves classification accuracy, achieving 90.15% and 90.64%, respectively.

Current research on the diagnosis of Schizophrenia by ML and DL has several significant limitations. Numerous methods suffer from small or sparse datasets, which limit the model’s generalizability and diminish its resilience across various populations. The complex, multimodal nature of Schizophrenia might not be adequately represented by particular models that rely on single-modality inputs like EEG, fMRI, miRNA, or ECG data. Despite frequently attaining great accuracy, DL architectures are less feasible for clinical implementation due to their large computing resource requirements. EEG-based techniques are especially vulnerable to signal noise and inter-individual variability, which compromises their consistency and reproducibility. **Table 2** summarizes the related work for Schizophrenia.

**Table 2:** Related work summary.

References	Dataset	Model/Techniques	Results	Limitations
Lalawat et al. (24)	fMRI connectivity matrices	Deep learning + connectivity analysis	Enhanced diagnostic precision using synergy of modalities	Limited to fMRI modality, high computational cost
Yang (25)	EEG signals	Fuzzy deep learning model	Effective diagnosis with uncertainty modeling	Lack of clarity in fuzzy logic interpretability
Sarwer et al. (26)	EEG data (Alzheimer + Schizophrenia)	Deep learning on functional connectivity	Distinguishes AD and SCZ with high accuracy	Limited generalizability across neurological conditions
ksikazek et al. (27)	RR intervals (ECG)	Deep learning on heart rate data	Promising results for SCZ/BP classification	ECG-based diagnosis less validated clinically
Heda et al. (20)	miRNA expression data	ML classifiers on miRNA features	Identified miRNA signatures with diagnostic value by achieving accuracy 90.5%, precision 89.3% and F1-score 90.1%	Limited by lack of multimodal data
Norouzi et al. (19)	Clinical patient records	ML models (SVM, RF, etc.)	Accurate (93%) classification of schizophrenia vs controls	Limited generalization due to small dataset
Weng et al. (21)	Multi-site MRI data	Deep learning with interpretability methods	High performance and interpretable models	Requires extensive computation and crosssite harmonization
Srinivasan & Johnson (22)	Multi-channel EEG signals	Preprocessing + Deep learning (CNN)	Improved detection accuracy with optimized pipelines	EEG data prone to noise and variability
Xu et al. (23)	fMRI time- series data	Spatio-temporal residual GCN	High precision in automated diagnosis	Complex architecture may hinder clinical translation
Bhadra et al. (28)	Multiview EEG datasets	Optimized DL on multiview signals	Outperformed traditional EEG- based classifiers	Model optimization and reproducibility concerns

## 3 Materials and methods

**Figure 1:**
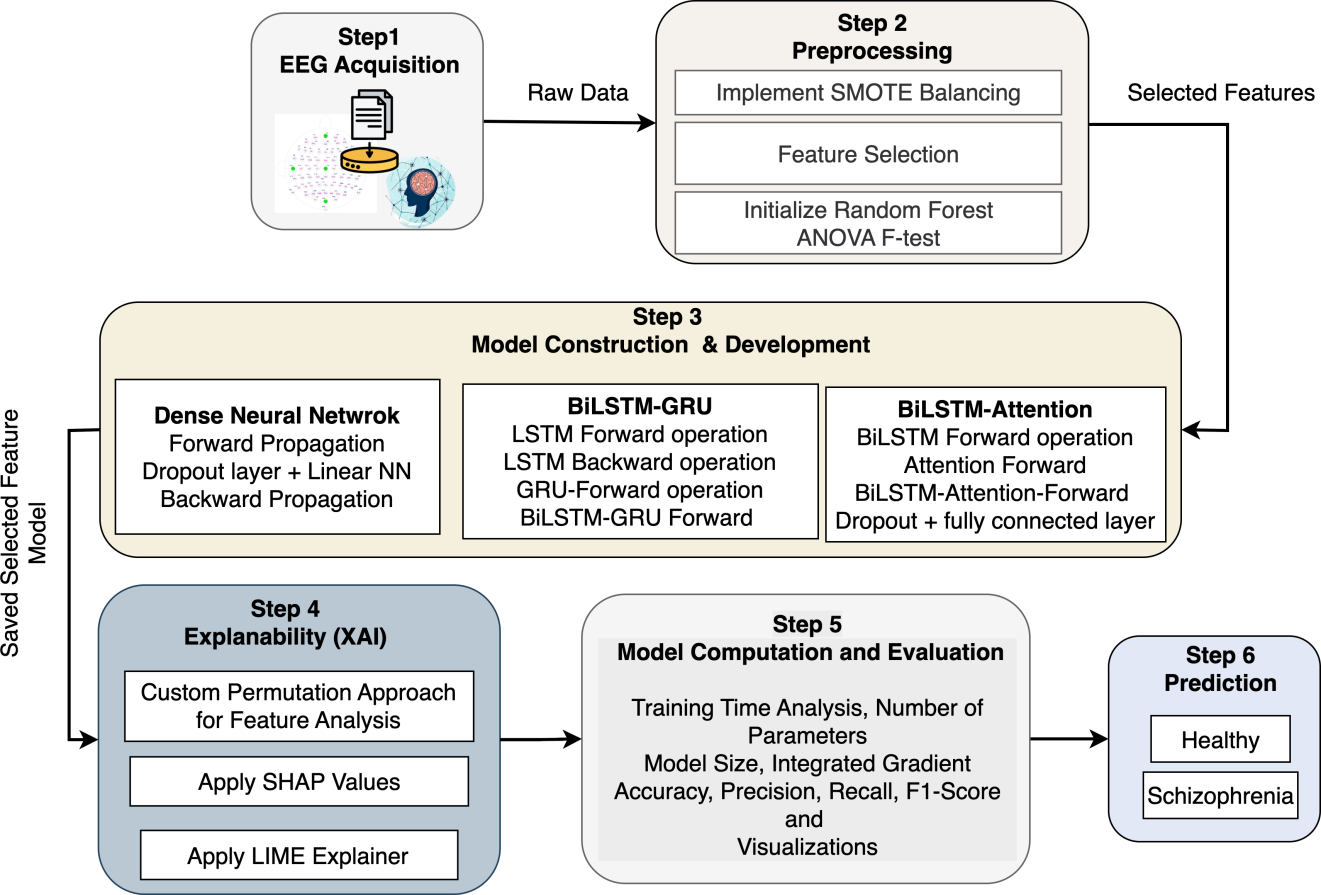
Proposed architecture for schizophrenia detection.

The proposed architecture provides a comprehensive and computational basis for diagnosing Schizophrenia using EEG data and advanced machine learning (ML) techniques, such as the XAI tools in **Figure 1**. Step 1 (EEG Acquisition) collects raw EEG signals as multivariate time-series data with *N_e_* electrodes and *T* time points. To distinguish between individuals with Schizophrenia and healthy controls, the data on cerebral electrical activity are significant. SMOTE is used in Step 2 (Preprocessing) to filter out noise and address the issue of class imbalance. It performs this by interpolating between nearest neighbors in feature space to create fresh samples ${\hat{x}}_{j}\in {\mathbb{R}}^{d}$ for the minority class. The ANOVA F-test statistic and Random Forest significance scores are used for feature selection. For feature *f_i_*, *MSB_i_* and *MSW_i_* represent the mean squares between and within groups, respectively. Thus, $X^{\prime }\in {\mathbb{R}}^{n\times d^{\prime }}$ , where $d^{\prime }<d$ , is a reduced feature matrix. Three deep learning models are created and trained on $X^{\prime }$ with corresponding labels in Step 3 (Model Construction & Development). The activation occurs at layer *l* of the DNN model, which is built of several completely connected layers. To analyze sequential EEG data forward and backward and capture both short-term and long-term dependencies, the BiLSTM-GRU model combines LSTM units with GRUs. By calculating attention weights, the BiLSTM with Attention model improves this by concentrating on informative time steps in the EEG series. In Step 4 (Explainability), model interpretability is accomplished by estimating the marginal contribution of each feature *f_i_* to a prediction using SHAP values *ϕ_i_*, which are obtained using cooperative game theory. Using a sparse linear model $g\left(z^{\prime }\right)\approx f\left(z\right)$ , LIME provides instance-level interpretability by approximating local model behavior. When individual features are permuted, the model performance drops Δ*L*(*f_i_*) and is evaluated using a specific permutation importance approach. Furthermore, Integrated Gradients provide an alternative viewpoint on the relevance of input features by computing the attribution score. The DNN is notable for its low complexity (*θ* ≈ 3.8 × 10^3^) and short training time (*τ* ≈ 3.7 minutes). Still, the BiLSTM-Attention model delivers better accuracy and interpretability despite its larger complexity (*θ* ≈ 85 × 10^3^). The ideal trained model $\hat{f}:X^{\prime }\to \left\{0,1\right\}$ is then used to unseen EEG data in Step 6 (Prediction) to identify individuals as either “Healthy” or “Schizophrenia”.

### 3.1 Dataset description and preprocessing

The EEG Data from the Basic Sensory Task in the Schizophrenia dataset is a publicly available resource developed to make it easier to compare the brain responses of individuals with Schizophrenia to those of healthy controls. EEG recordings made during a sensory task that involved pressing buttons and listening to sounds are included in the dataset, which Brian Roach and collaborators gathered. This setup aims to investigate event-related potentials (ERPs), with a focus on the N1 and P2 components, which are crucial for understanding sensory processing impairments associated with Schizophrenia. **Table 3** provides a comprehensive overview of the EEG dataset used to identify Schizophrenia. The dataset includes EEG recordings from 81 individuals, 32 of whom are healthy controls and 49 of whom have been diagnosed with Schizophrenia. The gender distribution shows a potential gender gap that may limit the generalizability of the model’s results, with 67 men and 14 women. EEG patterns and cognitive responses may be impacted by the average participant age of 39 years and the mean educational attainment of 14.5 years. EEG data were collected using 64 scalp electrodes and 8 external electrodes using the BioSemi ActiveTwo system, which provides excellent spatial resolution. The temporal resolution of the signals, which were captured at 1024 Hz, was adequate to capture rapid neural transitions. An auditory tone was played after a button was pressed in a straightforward exercise intended to elicit particular event-related potential (ERP) components from the participants. The analysis concentrates on the N1 (80–100 ms) and P2 (150–190 ms) post-stimulus ERP components, which are recognized for their importance in the study of Schizophrenia and early sensory processing. The Fz, FCz, and Cz electrodes, which are frequently used to track fronto-central brain activity, are among the key electrodes examined.

**Table 3:** Dataset overview.

Attribute	Description
Total Subjects	81 (49 with Schizophrenia, 32 healthy controls)
Gender Distribution	14 females, 67 males
Average Age	39 years
Average Education	14.5 years
EEG Channels	64 scalp electrodes + 8 external electrodes (BioSemi ActiveTwo system)
Sampling Rate	1024 Hz
Task	Button press followed by auditory tone
ERP Components Analyzed	N1 (80–100 ms), P2 (150–190 ms) post-stimulus
Electrodes of Interest	Fz, FCz, Cz
Data Size	Approximately 8.2 GB

The preprocessing procedure for EEG data included multiple essential phases to ensure high-quality input for classification models designed to detect Schizophrenia. EEG recordings from two directories, which provided data on individual subjects, were first retrieved. These recordings included 9216 readings per trial across specific electrodes. Every *N* consecutive row (*n* = 16) in the EEG matrix *a* was averaged using a custom function called averaged_by_N_rows(*a,n*). This reduced temporal resolution does not sacrifice core signal dynamics. The data was smoothed, and this downsampling significantly reduced its dimensionality. To compute the reshaped matrix *b*, the original matrix *a* was reshaped into a new shape (*a.*shape[0]*,n,a.*shape[1]). By taking the mean over axis 1 (i.e., across every set of *n* rows), *b*_mean_ was obtained. The data was smoothed, and temporal resolution was greatly decreased without compromising essential features due to this downsampling procedure. After the initial matrix *a* was reshaped into a three-dimensional array of shape (*a.*shape[0]*,n,a.*shape[1]), the matrix *b*_mean_ was created by computing the mean along the second axis. In addition to these procedures, the preprocessing pipeline included essential artifact removal methods to eliminate noise from EEG recordings. To increase the robustness and comparability of the model, amplitude variations were also standardized across trials and patients using signal normalization techniques. These stages were followed by recording the associated diagnostic labels in vector *Y* and storing the flattened feature vectors from each processed trial in array *X*. By ensuring that the data input into classification models was clean and consistent, this thorough preprocessing approach supported more accurate schizophrenia identification.

#### 3.1.1 Synthetic minority over-sampling technique

The dataset exhibited a large class imbalance since the class distribution had more schizophrenia cases (*Y*) than controls. This was tackled by generating new samples for the minority class (controls) synthetically using the Synthetic Minority Over-sampling Technique (SMOTE). By mathematically interpolating between a minority class instance *x_i_* and one of its *k*-nearest neighbors *x_zi_*, SMOTE creates synthetic samples in a process described in Equation 1:


(1) $$x_{\text{new}}=x_{i}+\lambda \cdot \left(x_{zi}-x_{i}\right),$$

where *λ* ∼ *𝒰*(0,1) is a random integer selected from a uniform distribution, *x_i_* ∈ ℝ*^d^* is a minority class sample, and *x_zi_* ∈ ℝ*^d^* is one of its nearest neighbors. The feature space of the minority class is efficiently filled by the synthetic samples, which are guaranteed to lie along the line segments connecting *x_i_* and its neighbors, thanks to this interpolation technique. This enhances the classifier’s capacity for generalization and reduces the bias introduced by class imbalance.

#### 3.1.2 Feature selection

Three complementary techniques were employed to choose features: Random Forest importance, Mutual Information (*I*(*X,Y)*), and ANOVA F-test. For each technique, the top *k* attributes were selected based on scoring measures. For instance, the ANOVA F-test calculates the F-statistic (Equation 2).


(2) $$F=\frac{\text{Between}-\text{group variance}}{\text{Within}-\text{group variance}},$$

To assess feature importance. Mutual Information (MI) is computed as in Equation 3.


(3) I(X,Y)=H(X)−H(X|Y)

Where *H* stands for entropy and *H*(*X* | *Y)* is the conditional entropy between the target class and the characteristics. The Gini index, which measures the decrease in impurity that each feature contributes across all decision trees in the ensemble, thereby indicating its significance in classification tasks, was used to compute the Random Forest relevance.

### 3.2 Explainability and transparency

Artificial Intelligence, specifically machine learning (ML) and deep learning (DL) models, has shown promise in diagnosing Schizophrenia through the analysis of complex biomedical data, including genetic markers, MRI, fMRI, EEG, and ECG. A significant obstacle to incorporating AI algorithms into clinical practice is explainability, or the ability to understand and evaluate how these algorithms arrive at their conclusions. Explainable AI (XAI) approaches aim to make these “black-box” models more transparent and dependable for researchers and medical practitioners. To ascertain which parts of the brain, connection patterns, or signal properties have the most influence on diagnosis, techniques like SHAP (SHapley Additive exPlanations) and LIME (Local Interpretable Model-agnostic Explanations) have been employed.

#### 3.2.1 SHapley Additive exPlanations

SHAP is a unified approach for evaluating ML model predictions based on Shapley values from cooperative game theory. To explain the model’s output, *f*(*x*), it calculates the contribution of each input feature to the prediction. According to cooperative game theory, the feature *i*’s Shapley value *ϕ_i_* is defined in Equation 4:


(4) $$\phi_{i}\left(f,x\right)=\sum_{S\subseteq N\setminus \left\{i\right\}}\frac{\left|S\right|!\;\left(\left|N\right|-\left|S\right|-1\right)!}{\left|N\right|!}\left[f_{S\cup \left\{i\right\}}\left(x_{S\cup \left\{i\right\}}\right)-f_{S}\left(x_{S}\right)\right]$$

In this case, *N* represents the set of all input features, *S* represents a subset of features that lack the feature *i*, *f_S_* (*x_S_*) represents the model prediction using only features in subset *S*, *x_S_* represents the input feature values in subset *S*, and *ϕ_i_* represents the Shapley value, or the contribution of feature *i* to the prediction. SHAP belongs to the category of additive feature attribution techniques, which describes a model by (Equation 5):


(5) $$f\left(x\right)=\phi_{0}+\sum_{i=1}^{M}\phi_{i}$$

The model output for input *x* is *f*(*x*), and the base value is *ϕ*_0_. The predicted model output tends to be E[f(x)] . where *ϕ_i_* represents feature *i*’s contribution to the prediction. When diagnosing Schizophrenia, SHAP is used to assign model predictions to particular brain regions or biomarkers, especially when modalities like EEG data are used. Clinicians may gain a deeper understanding of which anatomical or functional features have the greatest influence on the model’s choice by utilizing SHAP’s interpretable outputs, which quantify the contribution of each input feature to the overall prediction.

#### 3.2.2 Local interpretable model-agnostic explanations

LIME is a method for deciphering predictions from black-box models by learning a simpler, interpretable model (such as a linear model) that locally approximates the complex model. Given a complex model *f*, LIME learns an interpretable model *g* that approximates *f* in the neighborhood of *x* to explain the prediction *f*(*x*) for a specific instance *x*. When *G* is the family of interpretable models, LIME formulates an optimization problem to identify the explanation model *g* ∈ *G* by minimizing (Equation 6):


(6) $$L\left(f,g,\pi_{x}\right)+\text{Ω}\left(g\right)$$

Simpler, more understandable explanations are encouraged by *L*(*f,g,π_x_*) si Local fidelity, which measures how closely *g* approximates *f* near *x*, *π_x_* (*z*), a proximity measure between *z* and *x* that weights the significance of occurrences around *x*, and Ω(*g*), the complexity of the explanation model. LIME employs a loss function such as squared loss given in Equation 7.


(7) $$L\left(f,g,\pi_{x}\right)=\sum_{z\in Z}\pi_{x}\left(z\right){\left(f\left(z\right)-g\left(z\right)\right)}^{2}$$

The collection of perturbed samples related to *x* is denoted by *Z*, the complex model’s prediction for sample *z* is *f*(*z*), the local surrogate model’s prediction is *g*(*z*), and a kernel function assessing similarity is *π_x_* (*z*). LIME was used to explain why an ML model classified an individual as neurotic by identifying the features (such as brain area sizes, EEG signal statistics, or connection metrics) that had the most considerable local influence on that specific prediction.

### 3.3 Deep neural network

A Deep neural network (DNN), also known as a fully connected network (FCN), is a type of artificial neural network in which all neurons in one layer are connected to all neurons in the layer above. Because DNNs can replicate complex correlations in data such as genetic, EEG, and neuroimaging signals, they are widely used for classification tasks, including schizophrenia diagnosis. DNN usually comprises several layers. Measurements of EEG signal intensities are examples of features that are received by the input layer. These layers’ neurons concentrate on the complicated relationships between input and output. The final layer generates the prediction, usually employing the class label (Schizophrenia vs. healthy) that is provided in **Algorithm 1**. The DNN can be expressed mathematically as (Equation 8) (29):

Algorithm 1 **Deep neural network model for Schizophrenia diagnosis.**

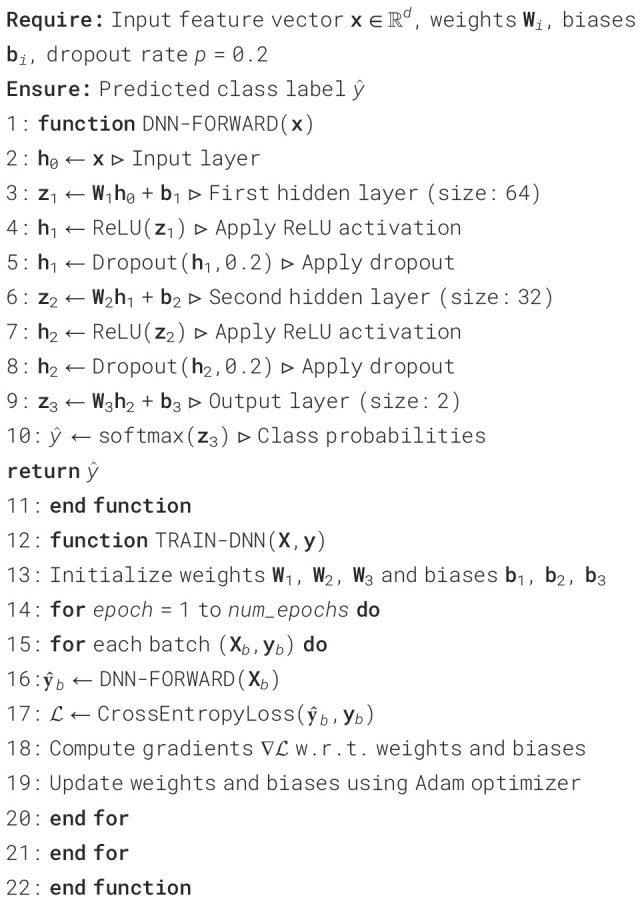

(8) $$h^{\left(l\right)}=f^{\left(l\right)}\left(W^{\left(l\right)}h^{\left(l-1\right)}+b^{\left(l\right)}\right)$$

With *h* ^(^ *^l^* ^)^ representing the *l*-th layer’s (activation) output, *W* ^(^ *^l^* ^)^ representing the *l*-th layer’s weight matrix, *b* ^(^ *^l^* ^)^ representing the *l*-th layer’s bias vector, *f* ^(^ *^l^* ^)^ representing the activation function (e.g., ReLU and Sigmoid), and *h* ^(^ *^l^* ^−1)^ representing the output from the previous layer (for the input layer, this is the original data). The final prediction (Equation 9) is generated using the output *h* ^(^ *^L^* ^)^ of the last layer *L*:


(9) $$\hat{y}=f^{\left(L\right)}\left(W^{\left(L\right)}h^{\left(L-1\right)}+b^{\left(L\right)}\right)$$

Where $\hat{y}$ represents the network’s prediction (such as the probability of Schizophrenia), *f* ^(^ *^L^* ^)^ is usually a sigmoid function for binary classification or a softmax function for multi-class classification. A crossentropy loss function (Equation 10) is frequently employed in a standard classification problem, like diagnosing Schizophrenia:


(10) $$L\left(y,\hat{y}\right)=-\sum_{i=1}^{C}y_{i}\log\;({\hat{y}}_{i})$$

Where *y* is the real label, *C* is the number of classes (Schizophrenia vs. healthy), and $\hat{y}$ is the predicted probability for each class. The loss for binary classification reduces to (Equation 11):


(11) $$L\left(y,\hat{y}\right)=-\left(y\log\;(\hat{y})+\left(1-y\right)\log\;(1-\hat{y})\right)$$

### 3.4 Long short term memory

Long Short-Term Memory (LSTM) is a type of recurrent neural network (RNN) designed to identify long-term dependencies in sequential input (30). To diagnose Schizophrenia, LSTM models are beneficial for analyzing temporal sequences from EEG recordings, where each time step comprises activity from multiple brain regions or sensors. The time series data input is represented as X = x_1_,x_2_ *,…*,x*_T_*, where *T* is the number of time steps and x*_t_* ∈ ℝ*^d^* is the feature vector at time step *t*. The LSTM processes the sequence to produce hidden states as H = h_1_, h_2_ *,…*, h*_T_*. The LSTM uses the following equations to update its internal states at each time step *t*: Initially, the data from the prior cell state should be erased, as decided by the forget gate. It is computed as Equation 12 (30):


(12) $$f_{t}=\sigma \left(W_{f}x_{t}+U_{f}h_{t-1}+b_{f}\right)$$

In this case, *σ* represents the sigmoid activation function, *x_t_* is the input at time *t*, and $h_{t-1}$ is the prior hidden state. The input gate then determines which additional data should be included in the cell state (Equation 13):


(13) $$i_{t}=\sigma \left(W_{i}x_{t}+U_{i}h_{t-1}+b_{i}\right)$$

This complements the candidate cell state, which is calculated as in Equation 14:


(14) $${\tilde{c}}_{t}=\tanh\;(W_{c}x_{t}+U_{c}h_{t-1}+b_{c})$$

The hyperbolic tangent function that aids in bounding the candidate values in this case is tanh. The candidate data and the prior state are then combined, modulated by the input and forget gates, to update the cell state according to Equation 15:


(15) $$c_{t}=f_{t}⊙c_{t-1}+i_{t}⊙{\tilde{c}}_{t}$$

To indicate element-wise multiplication while maintaining the data’s structure, use the operator ⊙. The output gate in Equation 16 uses the cell state and current input to identify the next hidden state:


(16) $$o_{t}=\sigma \left(W_{o}x_{t}+U_{o}h_{t-1}+b_{o}\right)$$

Finally, the hidden state is updated according to Equation 17:


(17) $$h_{t}=o_{t}⊙\tanh\;(c_{t})$$

Subsequent classification tasks, such as recognizing Schizophrenia from brain activity sequences, are then performed using this hidden state *h_t_*. The sigmoid activation function is *σ*, the hyperbolic tangent function is tanh, element-wise multiplication is indicated by ⊙, and trainable parameters *W*_∗_, *U*_∗_, and *b*_∗_ are learned during the training process in all of the equations above.

### 3.5 Gated recurrent unit

Gated Recurrent Units (GRUs) are an effective kind of recurrent neural network (RNN) that captures temporal dependencies and are easier and faster to train than LSTMs when diagnosing Schizophrenia using sequential neuroimaging or electrophysiological data (31). The update gate and the reset gate are the two gates that the GRU uses to update its hidden state at each time step *t*. These gates regulate the flow of information and mitigate the vanishing gradient issue that conventional recurrent neural networks (RNNs) often encounter. The update gate determines which aspects of the previous concealed state should be retained and how much should be updated with new information, as represented in Equation 18 (31):


(18) $$z_{t}=\sigma \left(W_{z}x_{t}+U_{z}h_{t-1}+b_{z}\right)$$

where *w_z_*, *U_z_*, and *b_z_* are trainable parameters, *z_t_* is the update gate vector, *x_t_* is the input at time *t*, $h_{t-1}$ is the prior hidden state, and *σ* is the sigmoid activation function. When determining the candidate concealed state, the reset gate (Equation 19) decides the amount of the past data to disregard:


(19) $$r_{t}=\sigma \left(W_{r}x_{t}+U_{r}h_{t-1}+b_{r}\right)$$

Where the trainable parameters are *W_r_*, *U_r_*, and *b_r_*, and the reset gate vector is *r_t_*. The previous hidden state that has been reset is used to calculate the candidate hidden state ${\tilde{h}}_{t}$ given in Equation 20:


(20) $${\tilde{h}}_{t}=\tanh\;(W_{h}x_{t}+U_{h}\left(r_{t}⊙h_{t-1}\right)+b_{h})$$

where ⊙ is the element-wise multiplication and tanh is the hyperbolic tangent function. A linear interpolation between the candidate hidden state and the previous hidden state is represented by *h_t_*, the ultimate hidden condition in Equation 21, which is the update gate’s control:


(21) $$h_{t}=\left(1-z_{t}\right)⊙h_{t-1}+z_{t}⊙{\tilde{h}}_{t}$$

### 3.6 Bidirectional-LSTM

Bidirectional Long Short-Term Memory (BiLSTM) networks are highly effective at identifying both past and future dependencies in the data, particularly when analyzing sequential neuroimaging data for the diagnosis of Schizophrenia (32). Identifying fragile temporal patterns associated with the condition necessitates this. BiLSTM is formed of two LSTM networks: processing the sequence from the past to the future using a forward LSTM. Processing the sequence from the future to the past using a reverse LSTM. The model is then provided with both preceding and succeeding context by concatenating the hidden states from both directions at each time step. This is especially helpful in clinical time series, where the interpretation of previous patterns of brain activity can be influenced by future context. At time step *t*, the forward LSTM pass in Equation 22 calculates the hidden state

${\vec{h}}_{t}$ by processing the input *x_t_*, the cell state ${\vec{c}}_{t-1}$ , and the prior forward hidden state ${\vec{h}}_{t-1}$ :


(22) $${\vec{h}}_{t}={\text{LSTM}}_{\text{forward}}\left(x_{t},{\vec{h}}_{t-1},{\vec{c}}_{t-1}\right)$$

Likewise, the backward LSTM pass reverse-processes the input sequence to calculate the hidden state ${\overset{\leftarrow }{h}}_{t}$ . The following cell state ${\overset{\leftarrow }{c}}_{t+1}$ and the following hidden state ${\overset{\leftarrow }{h}}_{t+1}$ are used (Equation 23):


(23) $${\overset{\leftarrow }{h}}_{t}={\text{LSTM}}_{backward}\left(x_{t},{\overset{\leftarrow }{h}}_{t+1},{\overset{\leftarrow }{c}}_{t+1}\right)$$

The outputs of the forward and backward LSTM passes are concatenated at each time step *t* to create the BiLSTM representation $h_{t}^{bi}$ , which incorporates contextual data from both directions as seen in Equation 24:


(24) $$h_{t}^{bi}=\left[{\vec{h}}_{t};{\overset{\leftarrow }{h}}_{t}\right]$$

### 3.7 BiLSTM-GRU

The BiLSTM and Gated Recurrent Unit (GRU) layers are incorporated in the BiLSTM-GRU model to efficiently capture intricate temporal patterns in EEG-based feature sequences for the classification of Schizophrenia. To handle the input feature vector x ∈ ℝ*^d^* as a time series, **Algorithm 2** initially transforms it into a sequence structure. The forward LSTM computes the hidden state h*_t_* and updated cell state **c** *_t_* using conventional LSTM gate operations, forget, input, and output, enabling the model to retain and update pertinent memory over time. Two LSTM units in the BiLSTM layer have opposing functions: the forward LSTM processes the sequence chronologically to generate hidden states ${\vec{h}}_{t}$ and cell states ${\vec{c}}_{t}$ . In contrast, the backward LSTM processes the sequence in reverse order to generate ${\overset{\leftarrow }{h}}_{t}$ and ${\overset{\leftarrow }{c}}_{t}$ , capturing future context. To create a bidirectional representation, the concealed states from both sides are concatenated at each time step:

Algorithm 2 **BiLSTM-GRU model for classification.**

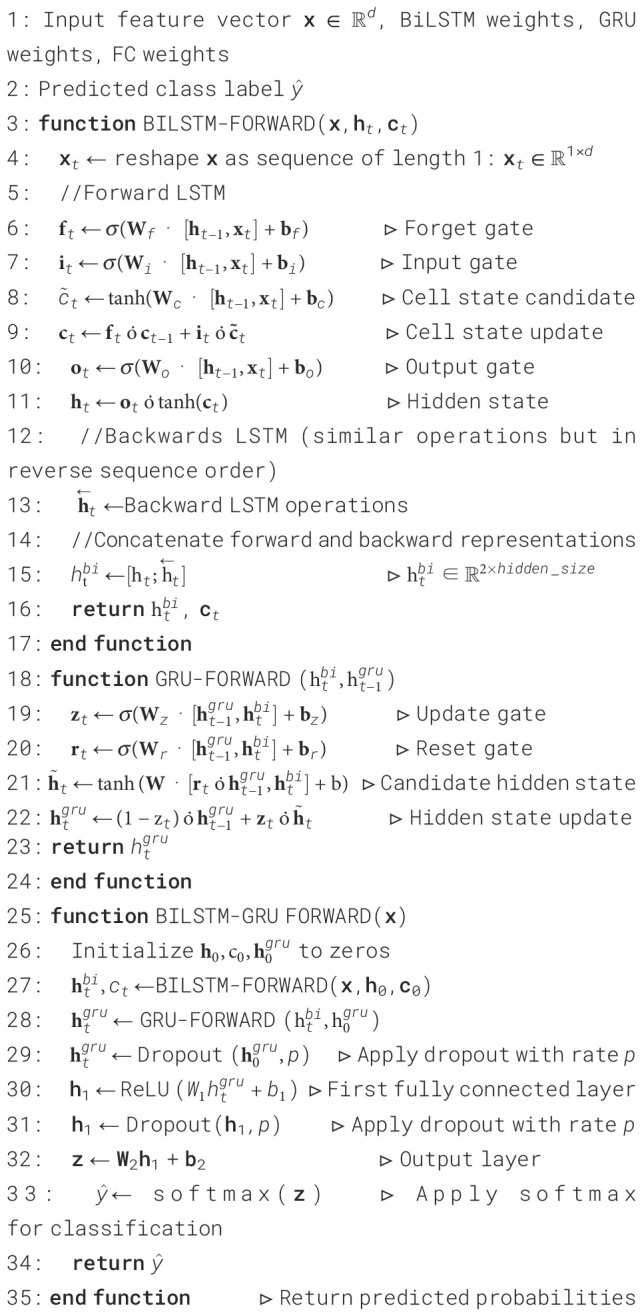

(25) $${\vec{h}}_{t}={\text{LSTM}}^{forward}\left(x_{t},{\vec{h}}_{t-1},{\vec{c}}_{t-1}\right)$$

(26) $${\overset{\leftarrow }{h}}_{t}={\text{LSTM}}^{backward}\left(x_{t},{\overset{\leftarrow }{h}}_{t+1},{\overset{\leftarrow }{c}}_{t+1}\right)$$

(27) $$h_{t}^{\text{bi}}=\left[{\vec{h}}_{t};{\overset{\leftarrow }{h}}_{t}\right]$$

This combined representation $h_{t}^{\text{bi}}$ is then sent into a GRU layer, which computes the hidden state $h_{t}^{\text{gru}}$ by further refining the temporal features through its update and reset gate processes. A fully linked layer with ReLU activation is used to introduce non-linearity, and dropout is used to lessen overfitting. The last Deep layer generates logits, which are then normalized using the softmax function to obtain class probabilities. Another dropout layer follows this. This architecture allows the model to classify Schizophrenia from EEG features with efficiency and resilience while utilizing both past and future temporal relationships.

### 3.8 BiLSTM with attention mechanism

The BiLSTM with Attention Mechanism is a potent deep learning architecture that was created to boost interpretability in addition to sequence classification performance (33). Bidirectional Long ShortTerm Memory (BiLSTM) networks’ contextual learning capabilities are combined in this model with a learned attention mechanism that draws attention to the most instructive segments of the input sequence. To determine which time steps have the greatest influence on the final classification decision, the attention mechanism gives weights to the hidden states, even if the BiLSTM component records temporal dependencies in both forward and backward directions. The approach provides more transparency into the decision-making process by explicitly modeling these attention weights, which helps practitioners and academics better comprehend and have confidence in the model’s predictions. Because interpretability is built into the model architecture, it is particularly well-suited for applications in sensitive disciplines such as neuroscience and healthcare, where understanding the model’s reasoning is crucial.

The model begins by processing the input feature vector **x** ∈ ℝ*^d^* through a BiLSTM layer as presented in **Algorithm 3**. The input undergoes forward and backward processing after first being rearranged into a sequence of length 1. This leads to a series of hidden states H ∈ ℝ^1×2^ *^h^*, where *h* is the size of the hidden state of the LSTM in one direction. Concatenating the final hidden states from both directions, ${\vec{h}}_{n}$ and ${\overset{\leftarrow }{h}}_{n}$ , produces the context vector $h_{n}=\left[{\vec{h}}_{n};{\overset{\leftarrow }{h}}_{n}\right]$ . The attention mechanism then uses the formula in Equation 28 (33) to determine each hidden state’s relevance score:

Algorithm 3 **BiLSTM with attention for classification.**

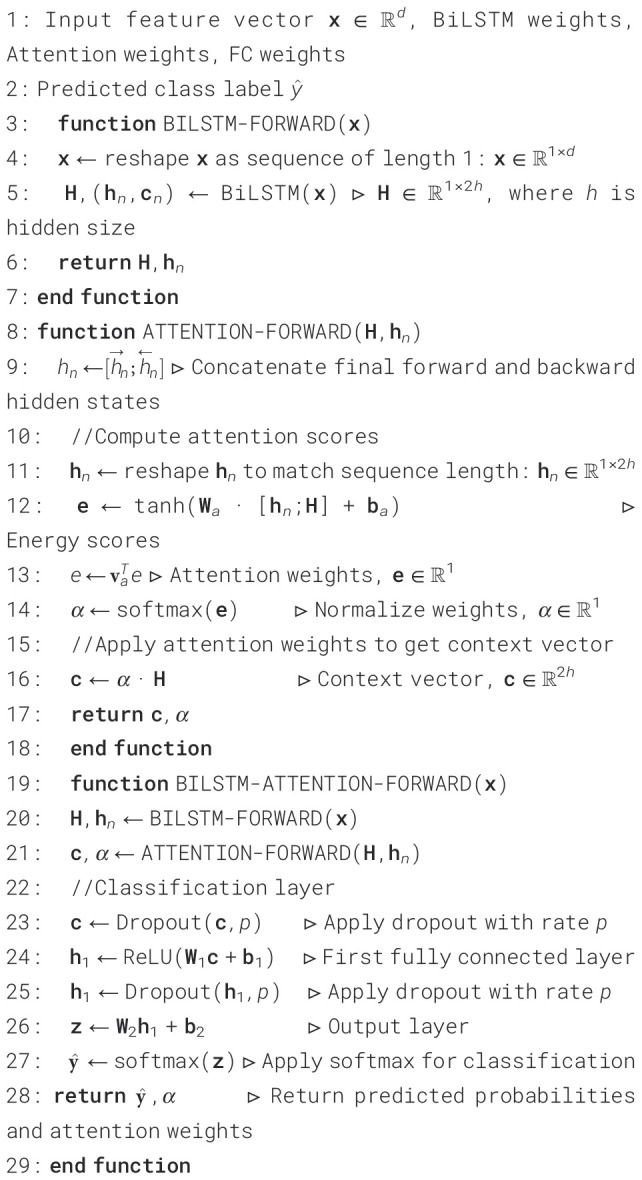

(28) $$e_{i}=v^{⊤}\tanh\;\left(W_{a}\left[h_{\text{i}};c\right]\right)$$

The context vector in this instance is **c**, the learned weight matrix is W*_a_*, the learned parameter vector is **v**, and the *i*-th hidden state is h*_i_*. The softmax function is then used to normalize these scores (Equation 29):


(29) $$\alpha_{i}=\frac{\text{exp}\;(e_{i})}{\sum_{j=1}^{T}\text{exp}\;(e_{j})}$$

The proportional importance of each time step is indicated by the attention weights *α_i_* that are produced. The attention context vector is calculated as a weighted sum of the hidden states using these weights (Equation 30):


(30) $$c_{\text{attn}}=\sum_{i=1}^{T}\alpha_{i}{\text{h}}_{i}$$

The sequence’s most instructive sections are combined in this vector **c**_attn_. To add non-linearity and lessen overfitting, it operates via a dropout layer and a fully connected ReLU layer. Before the final output layer, which uses the softmax function to generate class probabilities, a second dropout is performed. The classifier can thus focus on the necessary time steps in the input, thanks to the attention-enhanced BiLSTM model. This is particularly beneficial in fields such as EEG-based schizophrenia diagnosis, where specific signal segments may contain more potent diagnostic cues.

## 4 Experimental results and analysis

The effectiveness of the proposed deep learning architecture is evaluated using a wide range of assessment metrics, including training and testing time, model size, and model complexity, as provided in **Table 4**. To ensure repeatability and ease of experimentation, all models are developed and evaluated within the Google Colab environment. Google Colab offers a reliable and flexible cloud-based development platform featuring GPU acceleration, seamless Python integration, and access to powerful deep learning frameworks, including TensorFlow. The DNN is the fastest model in terms of training time, completing in approximately 3 minutes and 40 seconds. On the other hand, the LSTM requires the most time, taking more than 14 minutes to train in total. A similar pattern can be observed in the average epoch training time: the LSTM has the longest epoch duration, indicating that its sequential processing results in a higher computational cost. The LSTM is the slowest, requiring more than two minutes for assessment overall, while the DNN once again performs the best in terms of testing time, with the shortest average and total epoch testing lengths. The DNN is a lightweight model in terms of size, with just 3,874 parameters and a model size of 0.01 MB (15.13 KB). However, the BiLSTM-GRU has a massive amount of memory at 0.33 MB (337.88 KB) and the most parameters (86,498). Overall, the DNN is the most efficient in terms of size and time. Still, it lacks the expressive potential of attention-based and recurrent models, which compromise size and speed for greater learning capacity.

**Table 4:** Training time and model size statistics for different models.

Model	DNN	LSTM	BiLSTM-GRU	BiLSTM + Attention
Total Training Time	0:03:40.647739	0:14:22.014249	0:07:20.437193	0:05:43.926800
Avg Epoch Training Time	0:00:00.367746	0:00:01.436690	0:00:00.734062	0:00:00.573211
Total Testing Time	0:00:36.821652	0:02:03.156955	0:00:59.079024	0:00:46.524051
Avg Epoch Testing Time	0:00:00.061369	0:00:00.205262	0:00:00.098465	0:00:00.077540
Number of Parameters	3,874	50,562	86,498	72,002
Model Size	0.01 MB (15.13 KB)	0.19 MB (197.51 KB)	0.33 MB (337.88 KB)	0.27 MB (281.26 KB)

The benchmark for assessing performance is accuracy, which is the proportion of correctly identified samples relative to the total number of samples. Equation 31 shows the model’s accuracy, which indicates how confident people are in its capacity to generate precise predictions. Despite its ease of use, it is essential to evaluate its reliability and predictive capabilities.


(31) $$A=\frac{P_{\text{true}}+N_{\text{true}}}{P_{\text{true}}+N_{\text{true}}+P_{\text{false}}+N_{\text{false}}}$$

Precision is the degree of accuracy with which a model or system predicts the positive class. Equation 32 suitably displays this value to facilitate comprehension of the metric fundamental equation.


(32) $$Pre=\frac{P_{true}}{P_{true}+P_{false}}$$

Recall is a statistical measure used to evaluate the performance of classification algorithms, particularly in situations where detecting positive instances is crucial. It is sometimes referred to as the true positive rate or sensitivity. A model’s recall measures its ability to accurately distinguish genuine positive examples from all relevant occurrences, also known as true positives. The computation of Equation 33 demonstrates the special benefit of this varied viewpoint for an estimate.


(33) $$Re=\frac{P_{true}}{P_{true}+N_{false}}$$

Accuracy and recall are balanced by the appropriately calculated F1 score, which may effectively convey the essence of balanced performance. Although sophisticated, this simple estimation method is well described by Equation 34.


(34) $$F1-score=2\times \frac{Pre+Re}{Pre+Re}$$

### 4.1 DNN model results analysis

**Table 5** demonstrates the performance evaluation of a DNN model used to diagnose Schizophrenia. Class 1 indicates those who have been diagnosed with the condition, while Class 0 most likely indicates “Healthy” or “Non-schizophrenic” individuals. The precision of the model, which indicates the proportion of correctly predicted positive cases among all expected positives, is 0.70 for Class 0 and 0.65 for Class 1. This suggests that the model is better at identifying healthy individuals than people with Schizophrenia. Class 0 and Class 1 had recall values of 0.61 and 0.73, respectively, indicating that the model is better at identifying schizophrenia cases but misses more healthy individuals. For Class 0 and Class 1, the F1-score is 0.65 and 0.69, respectively, indicating a balanced trade-off. There were 1271 samples for Class 0 and 1270 samples for Class 1, which almost balanced the support values, which represent the number of real cases for each class. With an overall accuracy of 0.67, the model correctly identified 67% of all cases. Furthermore, the macro average, which accounts for all classes equally, produced precision, recall, and F1-scores of 0.67 each, demonstrating balanced performance independent of class frequency.

**Table 5:** DNN model performance.

Class	Precision	Recall	F1-score	Support
0	0.70	0.61	0.65	1271
1	0.65	0.73	0.69	1270
**Accuracy**			0.67	2541
Macro avg	0.67	0.67	0.67	2541
Weighted avg	0.67	0.67	0.67	2541

**Figure 2:**
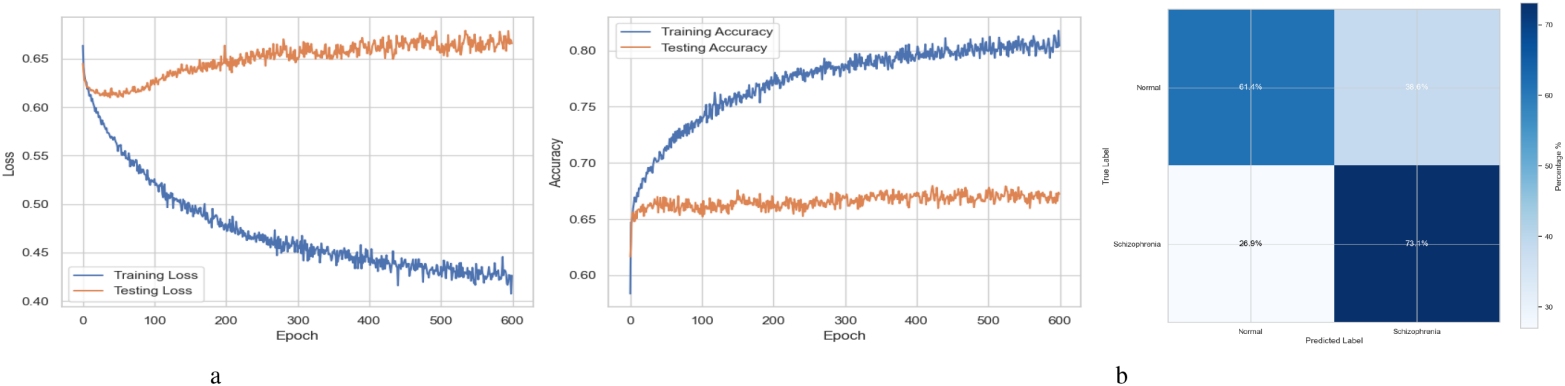
Performance visualization of the DNN model. **(a)** Training and validation accuracy and loss curve for DNN model. **(b)** Confusion matrix representation for DNN model.

**Figure 2a** summarizes the performance of a DNN throughout 600 training and testing epochs. The loss curve shows that the training loss gradually decreases, indicating effective learning, while the testing loss initially decreases, then plateaus, and subsequently increases slightly after approximately 100–150 epochs. Since the model continues to improve on the training data but struggles to generalize to new data, this pattern suggests that the model is overfitting to the training data. The accuracy curve is displayed across 600 epochs in the figure on the right. A steady improvement in training accuracy, approaching 80%, suggests that the training data is being effectively learned. Testing accuracy shows little increase after first increasing and plateauing at roughly 67-68% after 200 epochs. This trend indicates overfitting, as the model performs well on the training data but struggles to generalize its performance to new data. **Figure 2b** represents the confusion matrix of a DNN model used to diagnose Schizophrenia. The confusion matrix, which provides a visual comparison between the model’s predictions and the true labels, indicates that the model accurately diagnoses 61.4% of normal individuals (True Negatives) and 73.1% of instances of Schizophrenia (True Positives). The model sometimes misclassifies healthy people as having Schizophrenia and vice versa, as evidenced by its comparatively high false-positive rate of 38.6% and false-negative rate of 26.9%. Since incorrect diagnoses can have serious clinical repercussions, these misclassifications indicate that further model improvement is necessary.

**Figure 3:**
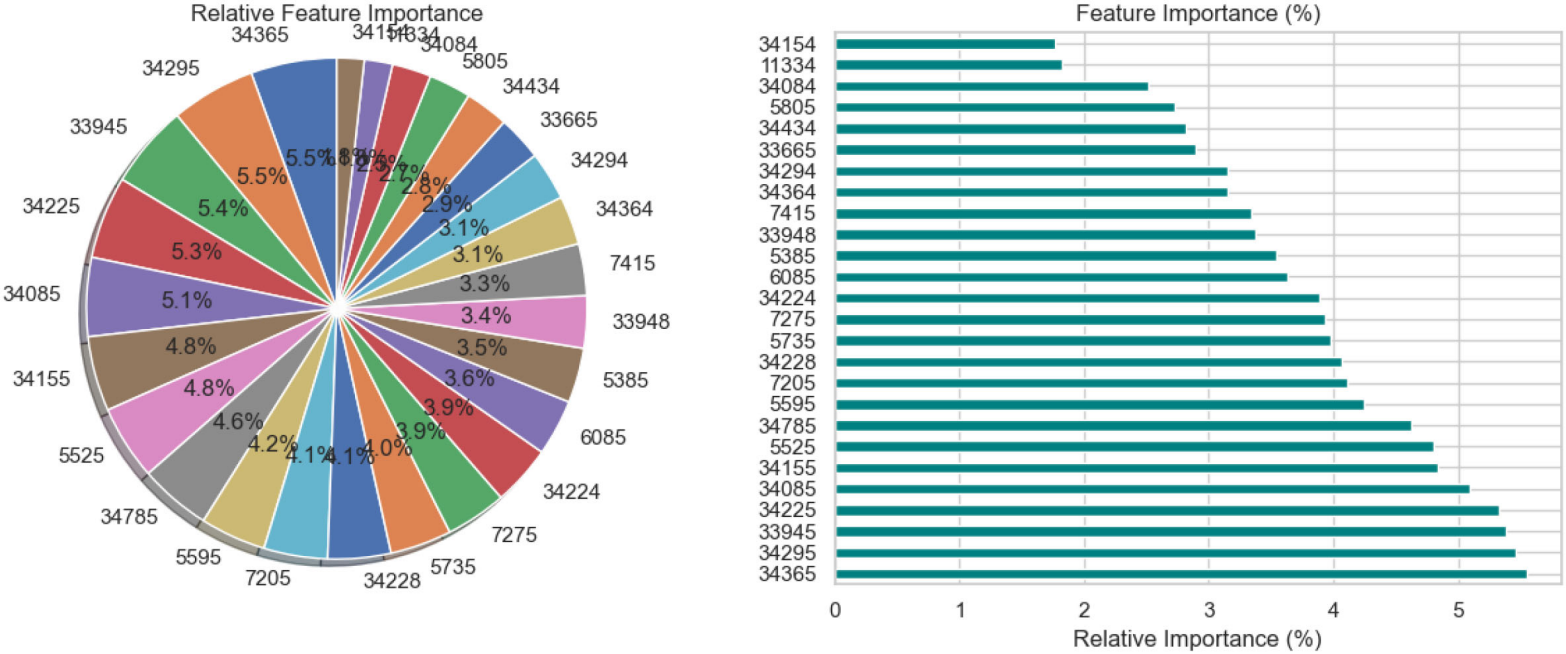
Relative feature importance analysis for DNN model in schizophrenia diagnosis.

The relative significance of the features that the DNN model uses to diagnose Schizophrenia is visually analyzed in **Figure 3** using a pie chart and a horizontal bar chart. The contribution of each feature as a percentage of the total is shown in the pie chart on the left, where larger slices represent more significant features. Interestingly, traits like 34295 and 34155 occupy a significant portion of the chart, suggesting that they are crucial to the model’s decision-making. By arranging the elements in descending order of significance and providing exact percentage values for each, the horizontal bar chart on the right enhances the visualization. For instance, feature 34295, which contributes 7.0% to the model’s predictions, is the most significant among the others with smaller percentages. These charts collectively demonstrate that although the model incorporates several features, only a small number of them are given significant weight. This data is necessary to comprehend the behavior of the model and can help direct feature selection strategies, especially when attempting to simplify the model or understand its predictions in a medical setting.

**Figure 4:**
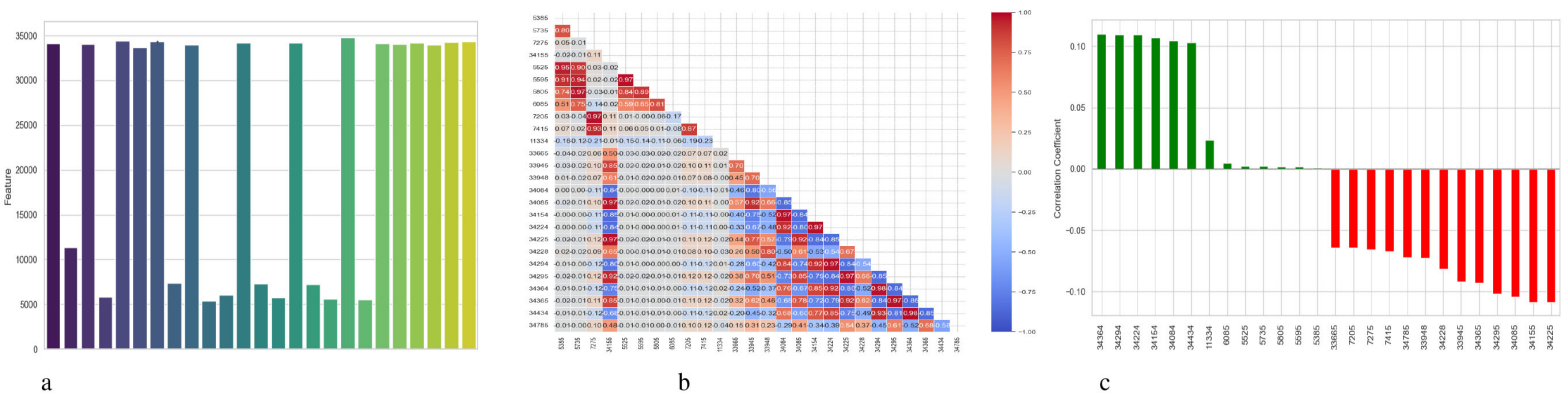
Feature correlation curves of DNN model for schizophrenia diagnosis. **(a)** Feature importance based on permutation. **(b)** Feature correlation. **(c)** Feature correlation with target variable.

**Figure 4** presents a comprehensive, three-part analysis of the features used by the DNN model to diagnose Schizophrenia. Permutation-based feature importance, inter-feature correlation, and feature correlation with the target variable have been included in this analysis. **Figure 4a** permutation-based bar chart illustrates the significance of each feature for the operation of the model. Features that significantly reduce accuracy when shuffled at random are considered very significant; these features manifest as tall bars, especially among feature indices between 0 and 5 and 14 and 17. However, because they contribute less to the predictive capacity of the model, shorter bars suggest characteristics that might be candidates for dimensionality reduction. In **Figure 4b**, the pairwise correlation between features is displayed using a triangular heatmap; high positive and negative correlations are represented by dark red and blue cells, respectively. Lightcolored cells suggest feature independence, and these patterns can highlight multicollinearity and feature redundancy. Finally, **Figure 4c** illustrates the linear relationship between each attribute and the target variable. Features that are most strongly linked to Schizophrenia are 34264 and 34154. Green bars show positive correlations, whereas red bars show negative correlations, such as those between traits 34755 and 344286. Although features with values close to zero exhibit inadequate linear correlations, they may still help identify non-linear patterns.

**Figure 5:**
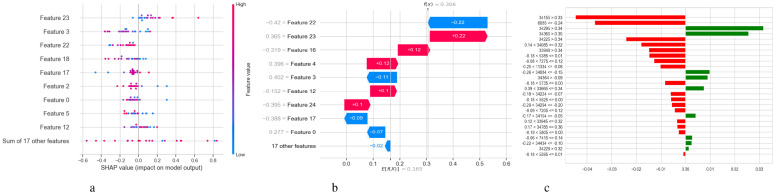
Model explanation using SHAP and LIME for DNN schizophrenia diagnosis. **(a)** SHAP values. **(b)** SHAP waterfall plot. **(c)** LIME explanation for predicted classes.

**Figure 5** illustrates two complementary methods for examining the predictions of a DNN model used to diagnose Schizophrenia: SHAP and LIME. **Figure 5a** displays a SHAP summary plot that shows that each feature affects the model’s output across the dataset. A row represents each feature, and each dot inside a row represents the SHAP value for a feature for a specific sample. The horizontal axis displays whether a factor influenced the prediction toward schizophrenia (positive SHAP value) or normalcy (negative SHAP value). Wide horizontal spread features are more influential since they exhibit a wider range of impact across events. For instance, “Feature 23”, “Feature 3”, and “Feature 22” exhibit significant variance, indicating that they have a substantial influence on the model’s decisions. Likewise, it is possible to identify the correlation between feature values and SHAP values; for example, high feature values may consistently raise the likelihood of Schizophrenia, indicating a positive association. A SHAP waterfall plot explaining the model’s output for a single prediction is shown in **Figure 5b**. It begins with a base value, which represents the average prediction for the entire dataset and illustrates how each attribute contributes to the final prediction for that specific instance. Red arrows point to features that improved the prediction score, while blue arrows point to features that made it worse. The magnitude of each arrow shows the strength of its contribution. For instance, with a SHAP value of +0.22, “Feature 22” significantly raised the prediction score, whereas “Feature 3” reduced it by -0.11. In **Figure 5c**, the LIME explanation for a single prediction appears as a horizontal bar chart. Whereas LIME builds a local linear surrogate model around the instance of interest, SHAP calculates accurate additive contributions. Features that either support (green) or contradict (red) the model’s classification choice are indicated by the bars. While the associated values display the feature’s real value in the instance, the length of each bar demonstrates the extent of the feature’s influence within this local model.

### 4.2 LSTM model results analysis

**Table 6** shows the performance characteristics of a Long Short-Term Memory (LSTM) model designed for diagnosing Schizophrenia. Class 0 probably represents the control group, which is made up of normal people, whereas Class 1 indicates those who have Schizophrenia. According to the per-class performance, 62% of the samples that were anticipated to be class 0 were correctly identified, and the model’s precision for class 0 was 0.62. With a recall of 0.65, it accurately identified 65% of real class 0 instances, yielding an F1-score of 0.64. The model’s accuracy, recall, and F1-score for class 1 were 0.63, 0.61, and 0.62, respectively. The support for classes 0 and 1 is almost identical, with 1271 samples in class 0 and 1270 in class 1. The accuracy of 0.63 indicates that the LSTM model successfully identified approximately 63% of the 2541 total cases, reflecting the model’s overall performance.

**Table 6:** LSTM model performance.

Class	Precision	Recall	F1-score	Support
0	0.62	0.65	0.64	1271
1	0.63	0.61	0.62	1270
Accuracy			0.63	2541
Macro avg	0.63	0.63	0.63	2541
Weighted avg	0.63	0.63	0.63	2541

**Figure 6:**
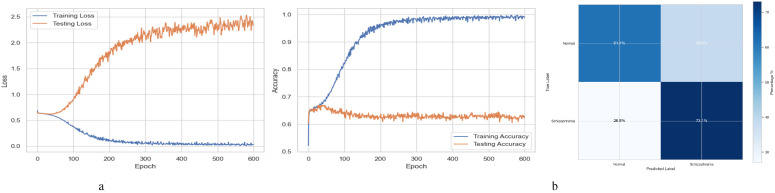
Performance visualization of the LSTM model. **(a)** Training and validation accuracy and loss curve for LSTM model. **(b)** Confusion matrix representation for LSTM model.

The training and testing curves in **Figure 6a** demonstrate the effectiveness of the LSTM model developed for the diagnosis of Schizophrenia. The training loss gradually approaches near-zero values after rapidly decreasing in the early epochs, as demonstrated by the loss curves on the left. This pattern demonstrates the model’s efficiency in detecting and recognizing the training data. However, the testing loss begins to rise between epochs 100 and 150 before fluctuating, suggesting significant generalization to new data. The accuracy curves are displayed in the graphic on the right. The training accuracy steadily increases to 99.99%, confirming the model’s outstanding fit to the training data. In parallel, the testing accuracy first increases before plateauing and exhibiting minor oscillations at approximately epochs 100 to 150, which is consistent with the behavior of the testing loss curve. The confusion matrix for the schizophrenia diagnosis LSTM model in **Figure 6b** indicates moderate classification performance. Detecting 60.7% of cases of Schizophrenia and 65.1% of normal individuals, the model showed an acceptable capacity to identify both classes. However, there were significant error rates, misclassifying 29.3% of patients with Schizophrenia as normal and 34.9% of healthy individuals as having Schizophrenia (false positives and false negatives, respectively). The estimated total accuracy for schizophrenia cases is 62.9%, with precision and recall of 63.5% and 60.7%, respectively. The model shows potential, but its high misclassification rates suggest that further improvements are needed to enhance its dependability in clinical applications.

**Figure 7:**
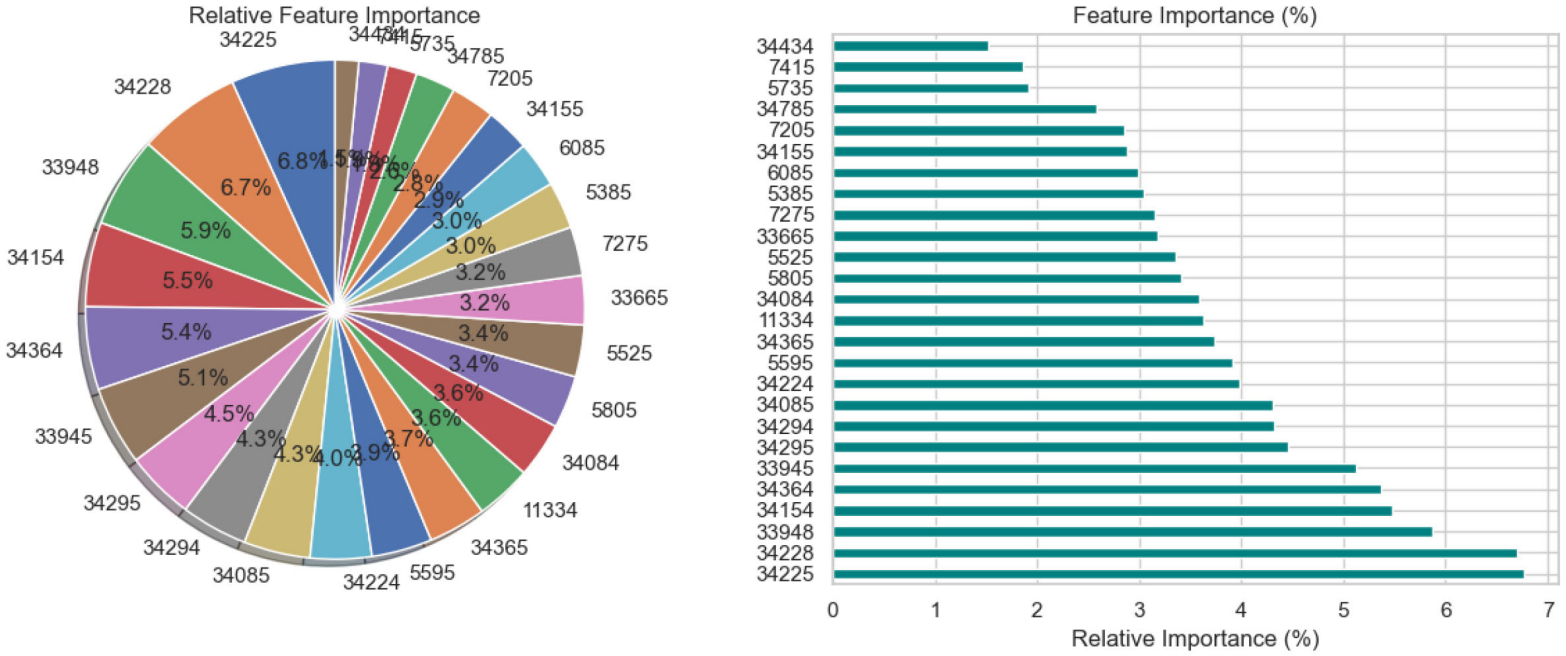
Relative feature importance analysis for LSTM model in schizophrenia diagnosis.

**Figure 7** depicts feature importance, providing crucial details on which input variables the LSTM model relied on most when predicting diagnoses of Schizophrenia. Both a pie chart and a horizontal bar chart are employed to illustrate the proportional contribution of each element. In both visualizations, longer bars or larger pie slices show that a characteristic has a bigger impact on the model’s decision-making. The most significant feature is ‘34225’, which is closely followed by ‘34228’ and ‘33948’. A few other features contribute minimally. The pie chart provides a comparative summary, while the bar chart facilitates easier comparison and ranking of feature relevance.

**Figure 8:**
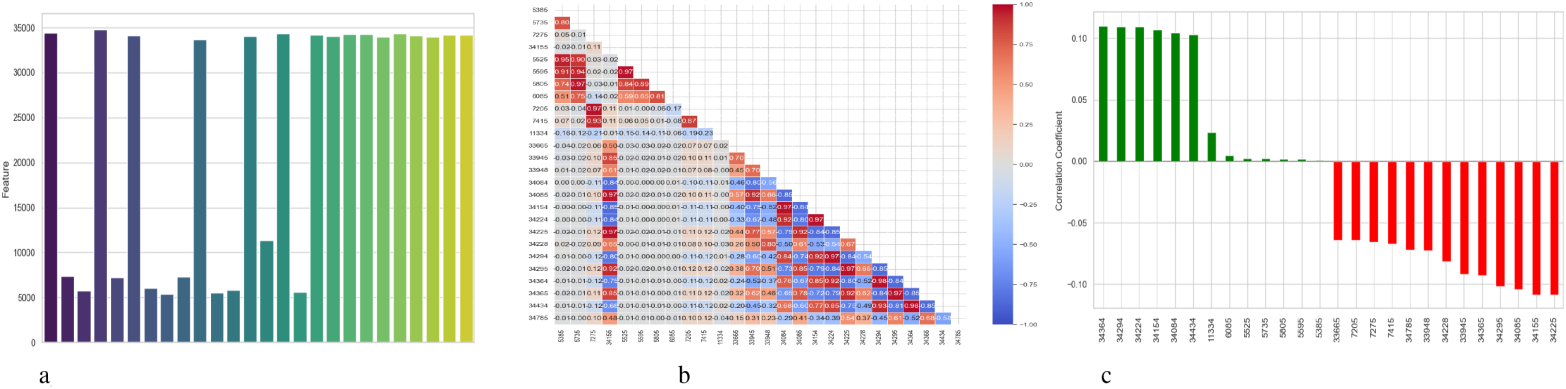
Feature correlation curves of LSTM model for schizophrenia diagnosis. **(a)** Feature importance based on permutation. **(b)** Feature correlation. **(c)** Feature correlation with target variable.

**Figure 8** provides a detailed analysis of the features used by the LSTM model to diagnose Schizophrenia using three subplots: permutation-based feature importance, pairwise feature correlation, and feature correlation with the target variable. Permutation-based feature importance is depicted in the first **Figure 8a**, where each bar indicates how significantly the model’s performance deteriorates when the values of a particular feature are randomly changed. Taller bar features are more crucial to the model’s expected accuracy, indicating that while many features have a minor effect, a few have a significant impact. This aligns with the commonly held notion in machine learning that a small set of features often holds the majority of predictive potential. A heatmap displaying pairwise relationships between features is the second subplot 8b. Clusters of features with strong interrelationships are seen in this illustration, suggesting possible redundancy where highly linked features can contain overlapping information. Potential feature reduction techniques that increase model efficiency without significantly compromising performance can benefit from recognizing such patterns. The final **Figure 8c** shows the linear association between each feature and the target variable, which is the diagnosis label for Schizophrenia. The height of each bar can determine the strength of the association. The features that are most linearly related to the diagnosis outcome are directly presented in this graphic, indicating which traits are more common in people with or without Schizophrenia.

**Figure 9:**
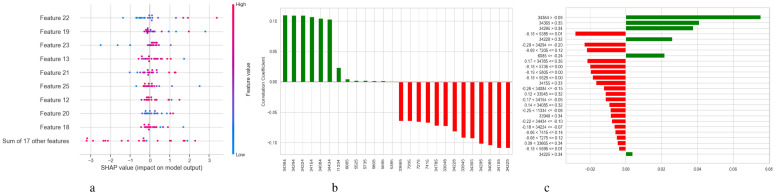
Model explanation using SHAP and LIME for LSTM schizophrenia diagnosis. **(a)** SHAP values. **(b)** SHAP waterfall plot. **(c)** LIME explanation for predicted classes.

**Figure 9** illustrates the analysis of an LSTM model’s predictions for schizophrenia diagnosis using SHAP and LIME. Each dot represents the SHAP value of a feature for a single instance in the SHAP summary plot depicted in Subplot 9a. Corresponding lists are shown on the y-axis, and their SHAP values are shown on the x-axis to demonstrate that features impact the model’s prediction. Positive values drive toward Schizophrenia, whereas negative values push toward a normal diagnosis. For instance, Feature 22 shows that higher values generally correspond to a higher risk of Schizophrenia. The distribution of dots along the x-axis for each property illustrates how significantly that feature influences distinct predictions. A thorough summary of each feature’s contribution to the LSTM model’s prediction is given for a single instance in the SHAP waterfall plot in subplot 9b. The SHAP value of each feature either increases or decreases the prediction, which is initially set to a base value (the model’s predicted output). Positive contributions are displayed by green bars, which raise the estimate, while negative contributions are indicated by red bars, which decrease the forecast. The cumulative effect that arises is the model’s result. This plot helps to understand not only which features were most influential but also the direction and size of those influences in that particular case. Subplot 9c displays the LIME explanation for the same or a different individual forecast at the end. LIME estimates the features that contributed most to the projected class by creating a simple interpretable model around the particular instance. Features and their values are displayed on the y-axis, while the x-axis displays the relative contributions of each feature to the categorization. The anticipated class (Schizophrenia) is supported by green bars and opposed by red bars. The feature has a larger impact on the forecast the longer the bar. In addition to the broad perspective provided by SHAP, this visualization offers an intuitive, local-level comprehension of the model’s reasoning for a particular case. When combined, these visuals improve confidence in the LSTM model’s diagnostic predictions and demystify its decision-making process.

### 4.3 BiLSTM-GRU model results analysis

**Table 7** provides a thorough analysis of a BiLSTM-GRU model’s performance, most likely when used for a binary classification classifying individuals as normal (class 0) versus diagnosing Schizophrenia (class 1). With a precision of 0.62 for class 0 (Normal), the model accurately identified 62% of the cases that were predicted to be normal. The model effectively detected 66% of real normal cases, as indicated by the recall of 0.66. The class’s overall performance is moderate, as indicated by the F1-score of 0.64, which maintains a balance between recall and precision. 1271 is the number of real instances. The precision for class 1 (Schizophrenia) is slightly higher at 0.64, indicating that 64% of the time, the model correctly identifies Schizophrenia. At 0.60, the recall is minimally lower, indicating that 60% of real cases of Schizophrenia are identified accurately. This class’s F1-score is 0.62, which once more indicates a moderate but balanced prediction ability. Class 1 support is 1270, indicating a dataset that is almost perfectly balanced. The model’s overall accuracy is 0.63, indicating that approximately 63.

**Table 7:** BiLSTM-GRU model performance.

Class	Precision	Recall	F1-score	Support
0	0.62	0.66	0.64	1271
1	0.64	0.60	0.62	1270
Accuracy			0.63	2541
Macro avg	0.63	0.63	0.63	2541
Weighted avg	0.63	0.63	0.63	2541

**Figure 10:**
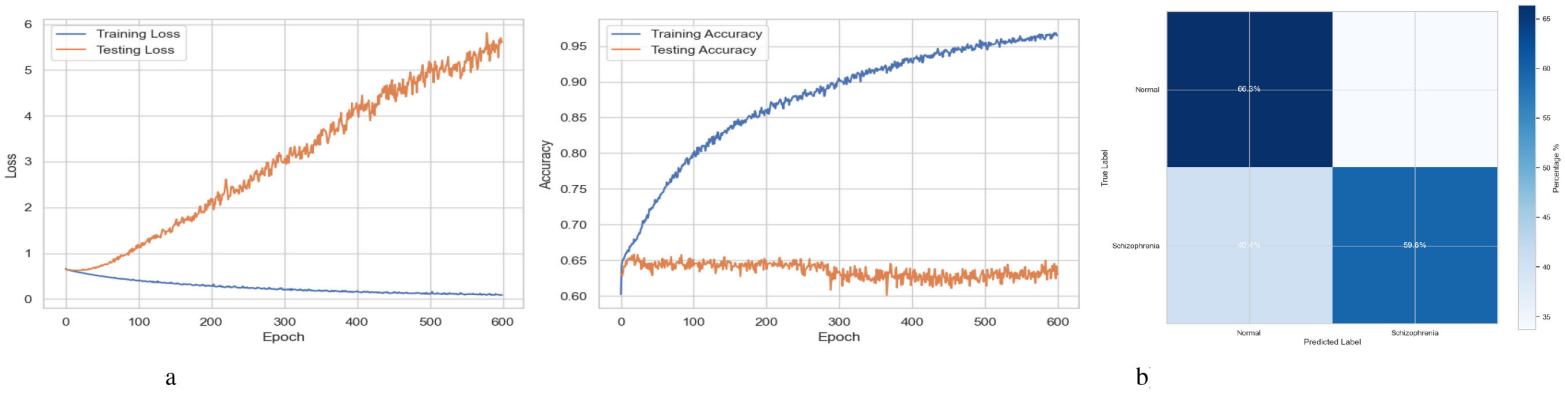
Performance visualization of the BiLSTM-GRU model. **(a)** Training and validation accuracy and loss curve for BiLSTM-GRU model. **(b)** Confusion matrix representation for BiLSTM-GRU model.

A BiLSTM-GRU model’s training and testing results over 600 training epochs are shown in **Figure 10a**. The loss value, which measures the discrepancy between the actual target labels and the model’s anticipated outputs, is displayed on the y-axis in the left subplot (Loss Curves). Lower loss values indicate better performance. The x-axis shows the number of epochs or complete runs of the training dataset. The precipitation loss, which starts high and progressively decreases, shows that the model is effectively learning from the training data and lowering prediction errors. The testing (or validation) loss initially exhibits a decreasing trend, comparable to the training loss, suggesting that the model is also improving at handling unobserved input. However, the testing loss starts to increase while the training loss continues to decrease after about 100 to 200 epochs. Training epochs are represented by the x-axis in the right subplot (Accuracy Curves), while accuracy, or the percentage of accurate predictions, is represented by the y-axis. Training accuracy, which steadily rises to about 95% by the conclusion of training. This indicates that the model is doing excellently in fitting the training data. The accuracy of the tests initially rises similarly. It starts to plateau, though, and then exhibits modest variations or even a slight fall around the same epoch range (100-200). **Figure 10b** displays a confusion matrix assessing a BiLSTM-GRU model’s performance on a binary classification by differentiating between “Normal” and “Schizophrenia” cases. The matrix shows that the model correctly identified 59.6% of individuals with Schizophrenia and 66.0% of “Normal” individuals. Additionally, it mislabeled 40.4% of actual cases of Schizophrenia as “Normal,” indicating a significant percentage of false negatives. However, the model performs slightly better in identifying non-schizophrenic individuals, which raises concerns given the potential clinical consequences of a missed diagnosis.

**Figure 11:**
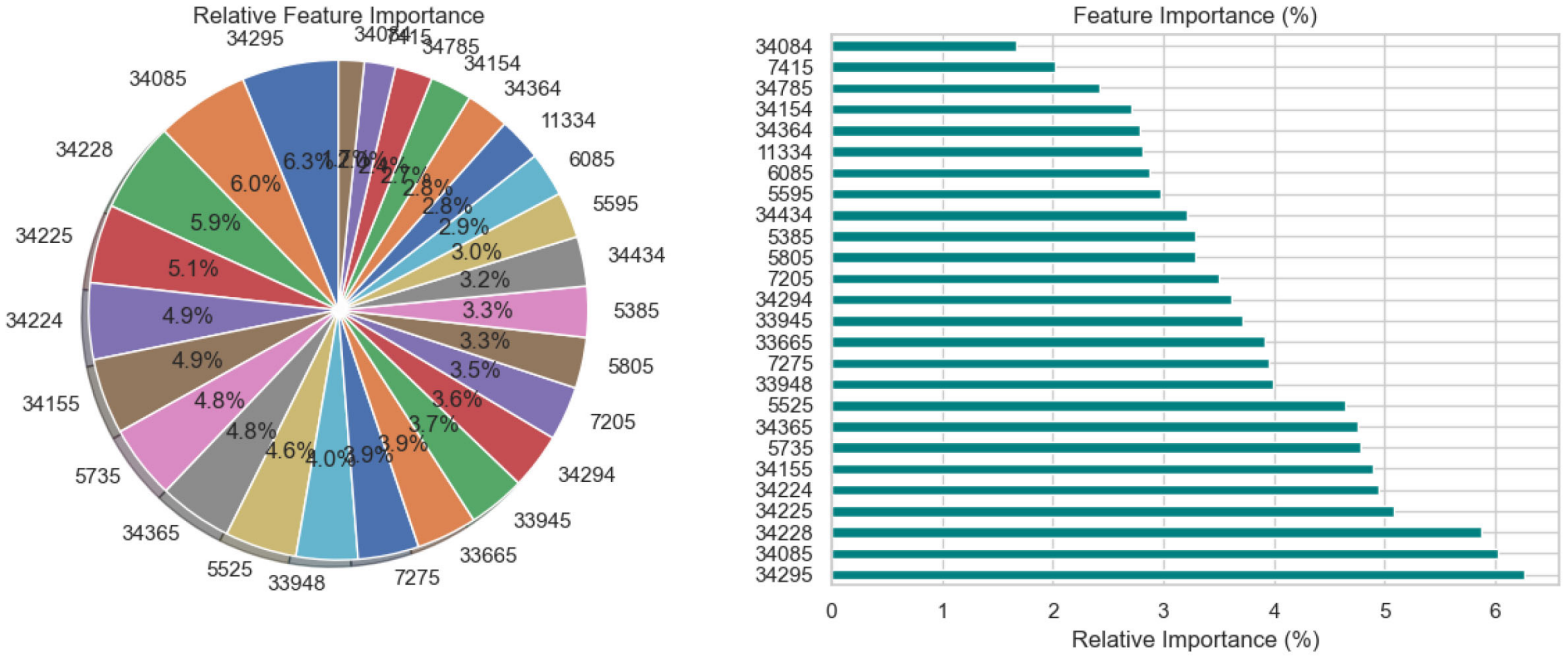
Relative feature importance analysis for BiLSTM-GRU model in schizophrenia diagnosis.

**Figure 11** provides a detailed analysis of the relative feature relevance for the BiLSTM-GRU model, which is used to diagnose Schizophrenia. The size of each slice in the pie chart on the left shows the magnitude of the contribution each feature made to the model’s predictions. Each slice is represented by a feature (identified by number codes such as 34295, 34085, etc.). For instance, the most significant slice (6.3%) of feature 34295 indicates that it has the most influence, followed closely by features 34085 and 34228, each of which contributes roughly 6.0%. The visual layout makes it easy to understand which features are most crucial to the model’s decision-making process. The same features are shown in descending order of significance in the right subplot, a horizontal bar chart, where the bar lengths represent the percentage contribution of each feature. When there are few variations in relevance, this format enables a more apparent and precise comparison of attributes. For instance, the bar chart more accurately identifies the top contributors and shows the slow drop in relevance among the remaining variables. In contrast, the pie chart graphically conveys that several features are significant.

**Figure 12:**
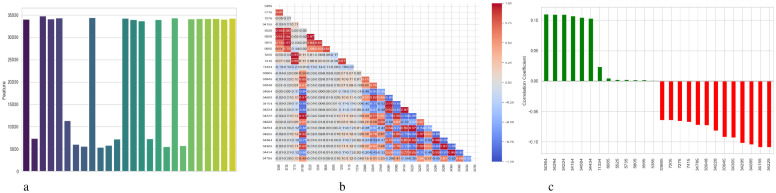
Feature correlation curves of BiLSTM-GRU model for schizophrenia diagnosis. **(a)** Feature importance based on permutation. **(b)** Feature correlation. **(c)** Feature correlation with target variable.

**Figure 12** provides a detailed assessment of the function and connections between the features that the BiLSTM-GRU model uses. Subplot 12a shows the feature importance obtained using a permutation-based approach, with the y-axis listing the features according to their numerical codes (e.g., 34295, 34085) and the x-axis representing the importance score. The importance of each feature to the model’s predictive accuracy is indicated by a vertical bar. The degree to which a feature’s values are randomly shuffled results in a decrease in the model’s performance, which is used to calculate the importance. A heatmap illustrating correlations between feature pairs is displayed in Subplot 12b, where lighter shades indicate weak or no correlation, blue indicates significant negative correlation, and red shows high positive correlation. It facilitates feature selection and dimensionality reduction by highlighting redundant characteristics that can offer overlapping information. Color-coded horizontal bars in Subplot 12c show the correlation between each attribute and the target variable, schizophrenia diagnosis. Positive correlations are shown by green bars, negative correlations by red bars, and the strength of the association is indicated by the length of each bar. The relevance of some features in model predictions can be demonstrated by the strong positive correlations they have with Schizophrenia, such as ‘34084’, and the strong negative correlations they have with Schizophrenia, such as ‘34225’.

**Figure 13:**
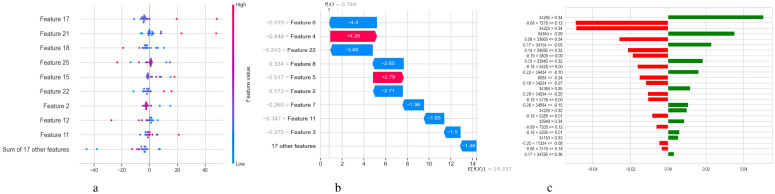
Model explanation using SHAP and LIME for BiLSTM schizophrenia diagnosis. **(a)** SHAP values. **(b)** SHAP waterfall plot. **(c)** LIME explanation for predicted classes.

**Figure 13** illustrates three visualizations that use SHAP and LIME to interpret the BiLSTM model’s predictions. With *x* ∈ R*^n^* being the input feature vector, these techniques aid in elucidating how input features *x_i_* contribute to the model’s output *f*(*x*). The SHAP summary figure is displayed in Subplot 13a, where the x-axis displays SHAP values *ϕ_i_*, which quantify the influence of each feature *x_i_* on the model output *f*(*x*), and the y-axis lists features (e.g., Feature 17, Feature 21). Color-coded by the feature value *x_i_* (red = high, blue = low), each dot represents a distinct case. Factors that push the model output toward a diagnosis of Schizophrenia are indicated by positive SHAP values *ϕ_i_ >* 0. In contrast, factors that push the output toward a normal diagnosis are indicated by negative SHAP values *ϕ_i<_* 0. The model’s decision-making is more influenced by features that exhibit a larger degree of variation in SHAP values among instances. A SHAP waterfall plot explaining the prediction *f*(*x*) for a single data instance is shown in Subplot 13b. The predicted model output across the dataset is represented by the base value E[f(x)] . The result is then shifted, either positively or negatively, by each feature *x_i_* and its matching SHAP value *ϕ_i_*. The sum of the SHAP contributions yields the final prediction. In this case, blue bars indicate positive contributions to the prediction (e.g., Feature 22 with *ϕ*_22_ = +3.86) while red bars indicate negative contributions (e.g., Feature 6 with *ϕ*_6_ = −4.4). The LIME explanation is presented in Subplot 13c, which locally approximates the BiLSTM model using a simpler linear model around the specific cases. The prediction is expressed as $f\left(x\right)\approx \sum w_{i}x_{i}$ , where *w_i_* is the local weight or importance of feature *x_i_* for the projected class. This contribution is shown by each horizontal bar, whose length indicates the contribution’s absolute magnitude |*w_i_x_i_* |. Red bars oppose the expected class (such as Schizophrenia), whereas green bars support it.

### 4.4 BiLSTM with attention results analysis

**Table 8** presents an extensive assessment of a BiLSTM model fitted with an attention mechanism that presumably predicts whether Schizophrenia would manifest (class 1) or not (class 0). The precision of 0.66 for class 0 in the model implies that 66% of the cases that were predicted to be class 0 were classified correctly, likely indicating cases of non-schizophrenia. With a recall of 0.73, the model identified 73% of true class 0 instances correctly. The F1-score of 0.70 indicates a balanced prediction performance for this class. Class 1 has a slightly higher positive predictive value than class 0, with a precision of 0.70. However, with a reduced recall of 0.62, the model missed more real class 1 occurrences. With an F1-score of 0.66, this performance is less balanced than that of class 0. There are about equal numbers of samples in each class, 1271 for class 0 and 1270 for class 1. With an overall accuracy of 0.68, the model demonstrated that 68% of all predictions were accurate. Without weighting by support, the macro average is likewise 0.68, indicating that the average performance is constant across the two classes. The weighted average, despite considering the distribution of the classes, likewise produces 0.68 for every metric, which is compatible with the roughly equal number of samples in the two classes.

**Table 8:** BiLSTM with attention mechanism model performance.

Class	Precision	Recall	F1-score	Support
0	0.66	0.73	0.70	1271
1	0.70	0.62	0.66	1270
Accuracy			0.68	2541
Macro avg	0.68	0.68	0.68	2541
Weighted avg	0.68	0.68	0.68	2541

**Figure 14:**
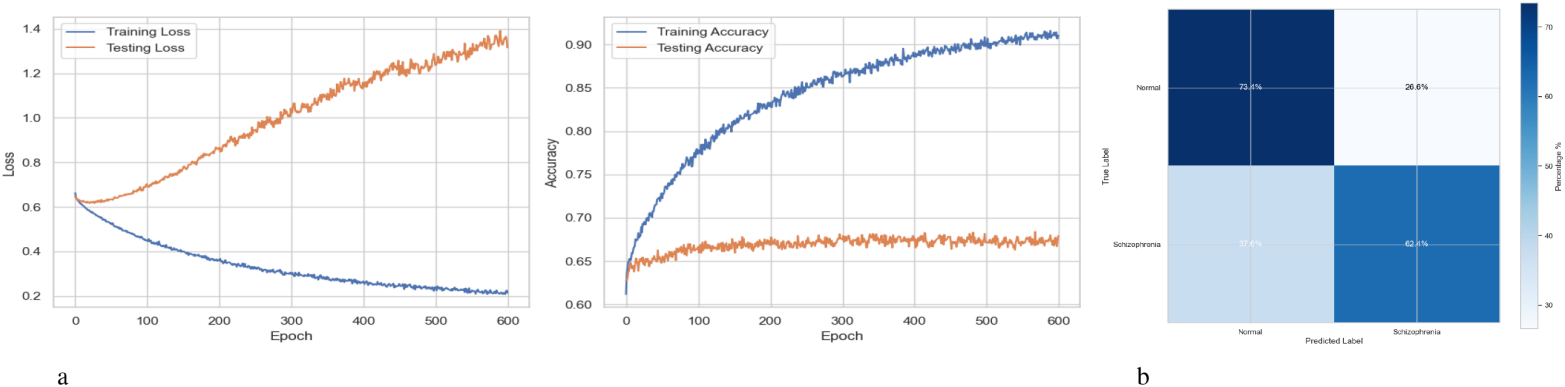
Performance visualization of the BiLSTM with attention mechanism model. **(a)** Training and validation accuracy and loss curve for BiLSTM with attention mechanism model. **(b)** Confusion matrix representation for BiLSTM with attention mechanism model.

**Figure 14a** illustrates the accuracy and loss curves in two subplots for the training and testing performance of a BiLSTM model with an Attention Mechanism across 600 epochs. When the model memorizes training data instead of generalizing, overfitting is indicated by the testing loss starting to rise around 50–100 epochs. Still, effective learning is indicated by the consistent reduction in training loss shown by the loss curves. While the training accuracy exceeds 90% in the accuracy curves, the testing accuracy plateaus or declines after approximately 50 to 100 epochs. **Figure 14b** depicts a confusion matrix evaluating the performance of a BiLSTM model with an Attention Mechanism, probably distinguishing between “Normal” and “Schizophrenia” individuals. The four cells in the matrix show the true positive, false positive, false negative, and true negative rates for each class. 73.4% of cases were accurately predicted by the algorithm for the “Normal” class, whereas 26.6% of cases were misclassified as “Schizophrenia.” The model properly predicted 62.4% of cases in the “Schizophrenia” class. However, 37.6% of cases were misclassified as “Normal.” With precision for “Normal” at roughly 66.13% and precision for “Schizophrenia” at roughly 70.11%, the inferred accuracy is 67.9%. With greater accuracy, recall for “Normal” (73.4% vs. 66.0%), recall for “Schizophrenia” (62.4% vs. 59.6%), and a reduced false negative rate for “Schizophrenia” (37.6% vs. 40.4%), the BiLSTM with Attention Mechanism outperforms a BiLSTM-GRU model.

**Figure 15:**
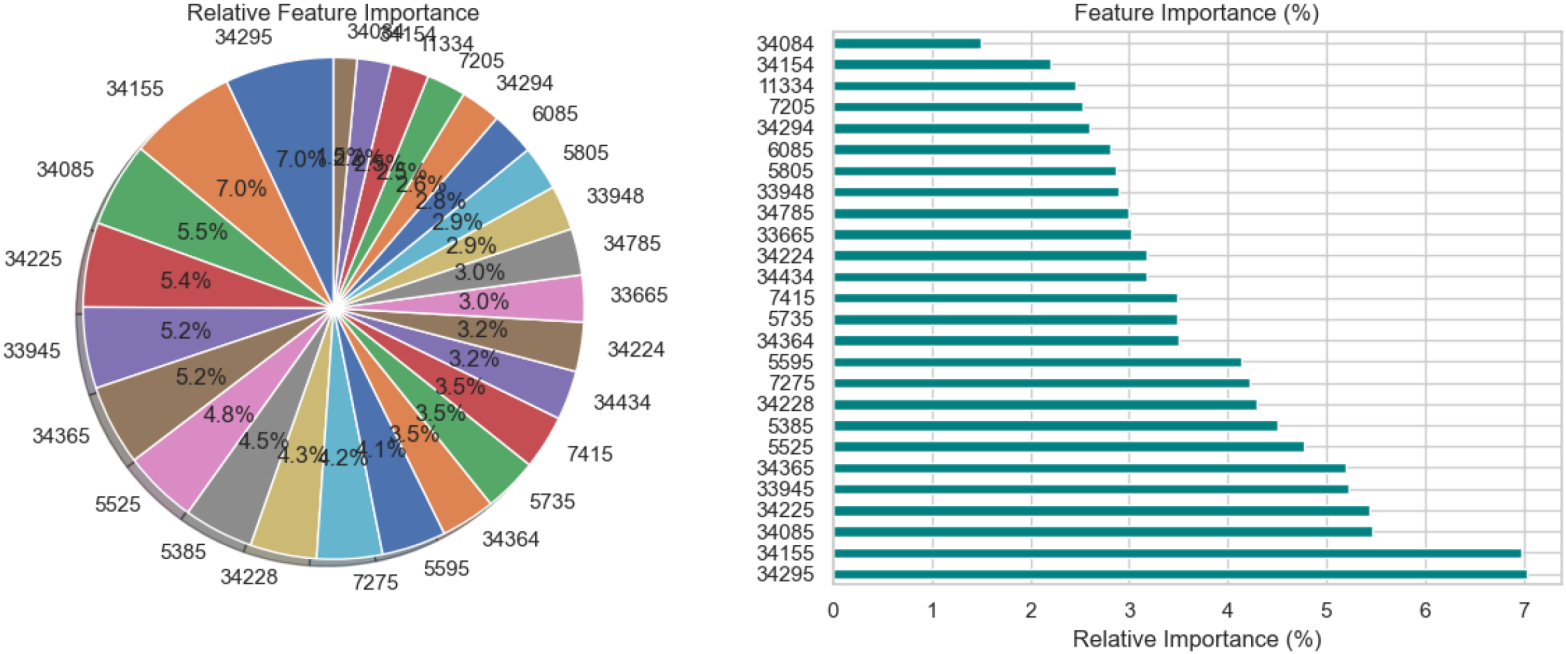
Relative feature importance analysis for BiLSTM with attention mechanism model in schizophrenia diagnosis.

The relative significance of the various features utilized by the BiLSTM model with an attention mechanism is depicted in **Figure 15**. To illustrate this significance, a pie chart and a horizontal bar chart are included. Larger slices indicate more significant features in the pie chart, which displays the percentage of each feature’s contribution to the model’s decision-making. For instance, with a contribution of 7.0%, feature “34295” is the most crucial, followed by “34155” and “34085.” This is enhanced by the bar chart, which provides a more accurate comparison by evaluating the qualities according to their significance, which facilitates the comparison of features of comparable value. Overall, the charts illustrate which features have a significant impact on the model’s predictions, with the most prominent features contributing more substantially than the others.

**Figure 16:**
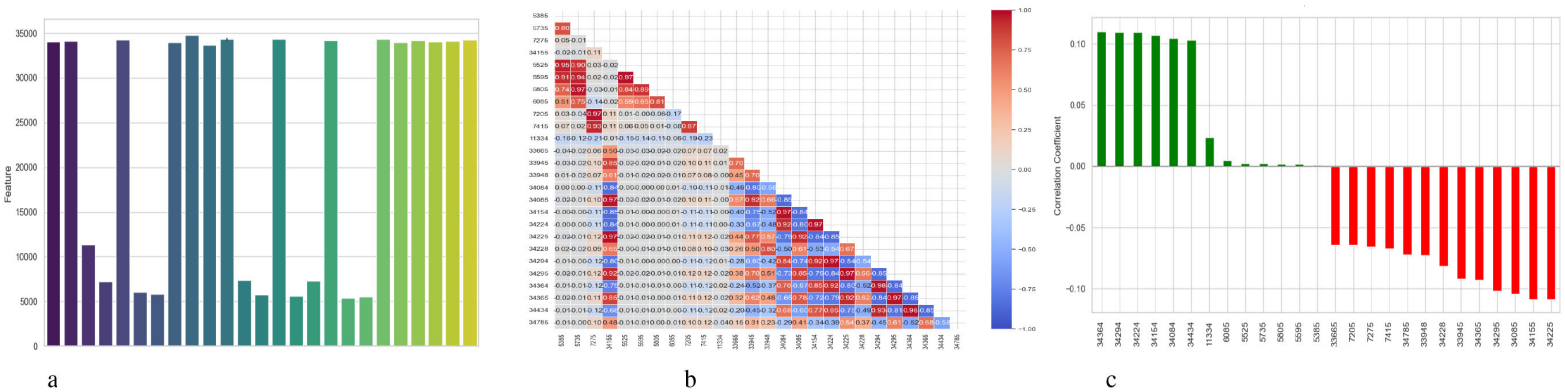
Feature correlation curves of BiLSTM with attention mechanism model. **(a)** Feature importance based on permutation. **(b)** Feature correlation. **(c)** Feature correlation with target variable.

**Figure 16** illustrates three evaluations related to the features used by the BiLSTM model with an attention mechanism. A feature’s relevance score is indicated by each vertical bar in the subplot 16a, which shows feature importance depending on permutation. While lower bar characteristics have a lesser effect, higher bar elements are more crucial for the model’s predictions. The correlation between features is shown in the subplot 16b using a heatmap; lighter hues suggest little to no link, blue denotes a negative correlation, and red denotes a positive correlation. This approach identifies redundancy between features, which can aid in feature selection and dimensionality reduction. The subplot 16c analyzes the relationship between the target variable (diagnosis of schizophrenia) and features. Whereas red bars show negative correlations, features with longer green bars have a positive linear association with the target. This subplot gives information on the predictive behavior of the model by highlighting the attributes that have the strongest correlations with the target variable.

## 5 Conclusion

This paper presents an extensive deep learning (DL) system for the automated detection of schizophrenia, utilizing EEG signals to enhance diagnosis accuracy and support treatment decision-making. By comparing and developing three architectures, the proposed framework demonstrates how attention-based techniques significantly enhance classification performance and interpretability. In addition to having the best accuracy of all the models evaluated, the BiLSTM-Attention model is a clinically valuable tool that provides insight into the neurophysiological mechanisms associated with schizophrenia. While the F-test feature selection increases computational efficiency and reduces the risk of overfitting, SMOTE effectively addresses the class imbalance in the dataset. Additionally, by identifying important EEG properties that contribute to predictions, the interpretability pipeline’s integration of SHAP and LIME enhances model transparency.

Despite the favorable outcomes, many limitations have to be addressed to contextualize the findings and direct future developments. Due to the small size of the study’s dataset, it may not accurately reflect the range of EEG patterns seen in different individuals, populations, and subtypes of schizophrenia. Due to this limited data scope, the model may not generalize effectively in larger or more diverse clinical contexts. Second, EEG data are naturally noisy and highly susceptible to aberrations from retinal squint, muscle movements, and environmental interference. Despite the use of preprocessing techniques to minimize noise, residual artifacts may still affect model predictions. Furthermore, the model’s capacity to acquire universally relevant properties may be limited by the notable inter-subject variability in EEG signal amplitude and spatial distribution. To enhance cross-subject robustness, future research should explore techniques such as domain adaptation and subject-independent modeling. Third, the attention weights and explanations generated by SHAP and LIME have not yet been clinically validated despite the BiLSTM-Attention model improving interpretability and outperforming baseline designs. It is unclear if the highlighted EEG findings represent significant neurophysiological patterns associated with schizophrenia in the absence of professional neurological input. There are issues regarding the framework’s generalizability because it has not been verified in various clinical contexts or with various EEG equipment. Furthermore, even if SMOTE corrects for class imbalance, the introduction of synthetic data may compromise model stability, underscoring the need for cross-subject and external validation. Finally, the real-world deployment of AI-based EEG diagnostic systems presents several challenges, including seamless integration into clinical workflows, ensuring that non-experts can utilize them easily, and managing fluctuations in data quality. Misdiagnosis, an excessive dependence on automation, and biases resulting from incomplete or artificial data are among the risks. Important ethical issues include data protection, informed consent, transparency, equity, and managing the psychological effects on patients through supervision and open communication.

Future research will focus on several key areas to enhance the clinical relevance, generalizability, and robustness of the proposed EEG-based schizophrenia detection framework, building on the encouraging findings of this study. To make the dataset more broadly applicable and minimize potential biases, it must first be expanded to include a larger number of participants from diverse demographic backgrounds and various clinical subtypes of schizophrenia. The model’s dependability under various technological circumstances will also be evaluated using validation across several EEG devices and recording locations. To increase diagnostic accuracy through deeper contextual awareness, multimodal data sources like neuroimaging, cognitive tests, and genetic information must be integrated. Future research should include clinical validation of the highlighted components to verify their alignment with established neurophysiological indicators and to gain new insights, despite the use of interpretability techniques such as SHAP, LIME, and attention mechanisms to highlight relevant EEG aspects. Additionally, utilizing methods such as model pruning and quantization, the model will be optimized for low power and real-time deployment, making it suitable for embedded and mobile EEG systems. Future developments will further enhance the development of customized models for patient longitudinal monitoring, enabling the tracking of treatment responses and early detection of relapse. To further enhance temporal pattern recognition and model expressiveness, improvements to the model architecture will be examined, including the addition of sophisticated attention mechanisms, such as multi-head or transformer-based attention. These approaches aim to enhance the practical application and therapeutic effectiveness of the suggested system.

## Data Availability

The original contributions presented in the study are included in the article/supplementary material. Further inquiries can be directed to the corresponding authors.
